# Comparative genomics of Clostridium butyricum reveals a conserved genome architecture and novel virulence-related gene clusters

**DOI:** 10.1099/mgen.0.001477

**Published:** 2025-08-12

**Authors:** Orlagh H. Anderson, James P. J. Chong, Gavin H. Thomas

**Affiliations:** 1 Department of Biology, University of York, Wentworth Way, York, YO10 5DD, UK

**Keywords:** chromid, *Clostridium butyricum*, pangenome, virulence

## Abstract

Bacteria from the species *Clostridium butyricum* encompass a diverse range of phenotypes. While some strains are used as probiotics, others have been isolated from cases of botulism and necrotizing enterocolitis (NEC) in preterm neonates. We identify a unique genomic feature of this species, namely a highly conserved extrachromosomal element of ~0.8 Mb. This replicon satisfies the three principal criteria used to define a chromid, which include the possession of core genes that are encoded on the main chromosome in other species. Although *C. butyricum* is the type species of *Clostridium*, we find that the possession of a chromid is not a typical feature of members of this genus and represents a unique genomic fingerprint of the species *C. butyricum*. Furthermore, we show that pathogenic *C. butyricum* strains from the sequenced examples are not monophyletic, which suggests that virulence has evolved multiple times from related non-pathogenic ancestors. However, we were able to identify common genes which are found exclusively in these pathogenic strains. In addition to the botulinum neurotoxin genes, these include a novel set of genes involved in the biosynthesis of a capsular polysaccharide (CPS), and genes that confer the ability to utilize the mucin-derived sugar l-fucose, which may provide a competitive advantage for growth in the colon. Moreover, by identifying NEC strain-associated virulence factors, we are able to further the understanding of these particularly harmful strains.

**Impact Statement**

Despite the rapidly expanding body of research into the potential industrial and biotechnological applications of *Clostridium butyricum*, our study represents the first comprehensive analysis of the genome architecture across the species. Although *C. butyricum* is the type species of the genus *Clostridium*, our study identifies a highly conserved chromid which is present in all sequenced *C. butyricum* strains, but absent from other members of the genus. Therefore, we demonstrate that the possession of this chromid can be considered a hallmark of *C. butyricum* and can be used to distinguish *C. butyricum* strains from other *Clostridium* species.

In addition to the beneficial applications of *C. butyricum* strains, several strains have been implicated in cases of infant botulism and NEC in preterm neonates. However, the mechanisms by which NEC-associated strains cause disease have been relatively understudied. In this study, we identify three novel gene clusters that are likely to be involved in the colonization and virulence of these strains. We therefore contribute towards the understanding of *C. butyricum*-associated NEC and provide a starting point for future studies on the treatment of this life-threatening condition. The genetic basis of the virulence factors identified in this study will also be important to consider during the genetic manipulation of *C. butyricum* strains for use as biotherapeutics.

## Data Summary

The authors confirm that the supporting data have been provided within the article or in the supplementary material.

## Introduction

The genus *Clostridium* is a highly heterogeneous group of Gram-positive, endospore-forming anaerobes [1]. The genus comprises over 200 species, which include the significant human pathogens *Clostridium botulinum* and *Clostridium perfringens*, as well as industrially relevant species such as *Clostridium acetobutylicum*. The genus *Clostridium* was proposed by Prażmowski in 1880 and *Clostridium butyricum*, which was initially isolated from pig intestines, was designated as the type species [2]. *C. butyricum* is commonly found in soil, cultured dairy products and as a gut commensal in both humans and animals [3,4]. It is often one of the first colonizers of the infant gut and can be found in the gut microbiome of ~20% of the adult population [4,6].

One of the main applications of *C. butyricum* is as a probiotic. The strain *C. butyricum* MIYAIRI 588 (CBM 588) was first isolated from a soil sample in Japan in 1963 and has been widely used in Asia as a commercially available probiotic for the treatment and prevention of diarrhoea, constipation and irritable bowel syndrome [7,9]. CBM 588 has also been approved for use in the European Union, as both an animal feed additive and a novel ingredient in food supplements [10]. It is thought that the primary mechanism by which probiotic strains exert their beneficial effects is through the production of short-chain fatty acids (SCFAs) from the fermentation of undigested dietary fibre [11,12]. A defining feature of *C. butyricum* is its ability to produce large quantities of the SCFA butyrate, which has proliferative effects on intestinal mucosal cells [13,15]. Butyrate also has immunomodulatory effects, which facilitate tolerance of other gut commensals and thus the prevention of pathogen overgrowth [16,20]. Probiotic *C. butyricum* strains have also been reported to act through the direct antagonism of enteropathogenic species through the production of bacteriocins and the inhibition of toxin production [21,26]. The properties of *C. butyricum* have also been investigated for potential therapeutic applications in a range of medical conditions, from colorectal cancer to depression [27,28]. Other novel uses of *C. butyricum* include a spore-based drug delivery system for the treatment of pancreatic cancer, and for the bioremediation of contaminated groundwater [29,30]. Like several other species of the genus *Clostridium*, *C. butyricum* also has applications in industrial biotechnology. *C. butyricum* can ferment crude glycerol to produce significant quantities of the industrially valuable chemical 1,3-propanediol, which is primarily used in polymer manufacture [31,33]. *C. butyricum* can also ferment various sugars and plant-based feedstocks to produce hydrogen and butanol for use as biofuels [34,37]. Less frequently, *C. butyricum* strains have been isolated from cases of botulism and necrotizing enterocolitis (NEC) in preterm neonates [38,42]. It has been well-established that botulism-associated strains cause disease via the atypical production of the botulinum neurotoxin type E (BoNT/E) [43,45]. The exact mechanism by which *C. butyricum* causes NEC is still unknown, although its development has been partially attributed to the production of neuraminidases and haemolysin-like polypeptides [46,48].

The first genome assembly project for a pathogenic *C. butyricum* isolate was completed in 2008 for the botulism-associated strain 5521 (GenBank accession no. ABDT00000000), while the first complete *C. butyricum* genome sequence was published in 2015 for the pit mud-derived strain JKY6D1 [49,50]. The genome sequence of the *C. butyricum* type strain DSM 10702 was first published as a contig assembly in 2013, and as a complete genome sequence in 2020 (GenBank accession no. AQQF00000000) [37]. As of January 2025, a total of 218 *C*. *butyricum* genomes at various levels of assembly have been deposited in the National Center for Biotechnology Information (NCBI) genome database.

To date, no studies have focussed on the genome architecture across the species *C. butyricum*. The only consideration of the genome organization in *C. butyricum* has been for ten BoNT/E-producing strains, which were isolated from clinical and environmental sources in China and Italy [51,53]. Using pulsed-field gel electrophoresis, it was determined that these ten neurotoxigenic strains each possess a large megaplasmid [51]. Moreover, using chemical treatment with acridine orange, Scalfaro *et al*. [53] were able to cure two strains of their megaplasmids [53]. Growth assays using these cured strains revealed that while loss of the megaplasmid can be tolerated under some growth conditions, it is essential for growth at the moderately low temperature of 15 °C and for the utilization of several carbon sources [53].

On the other hand, three groups have carried out extensive pangenome analyses of *C. butyricum*, using publicly available strains from the NCBI database and isolates from industrial sources, pit mud and mammalian stool samples [54,56]. All three studies find that the pangenome of *C. butyricum* is open, with phage sequences ubiquitous in the genomes, which suggests that frequent evolutionary events have occurred to allow adaptation to different environments. In line with this, it was found that up to 1,152 unique genes were identified per genome [54,56]. It was also found that *C. butyricum* strains isolated from the same niche are not necessarily phylogenetically related [54,56]. A further common finding is that the metabolic pathways which produce butyrate and 1,3-propanediol are largely conserved in the core or soft-core genomes (genes present in >99% or 95–99% of strains, respectively) [54,56]. In addition to the use of glucose and glycerol as carbon sources, it was found that the ability to utilize alginate, cellulose, galactooligosaccharides, isomaltooligosaccharides, pectin-galacturonic acid and trehalose is conserved across *C. butyricum* strains, while the ability to utilize xylose, xylooligosaccharides and inulin is only observed in a minority of strains [54,56]. Another well-documented hallmark of *C. butyricum* is its ability to fix atmospheric nitrogen into ammonia, which is subsequently assimilated into glutamate. Zou *et al*. [54] found that genes required for nitrogen fixation were present in 22 of their 24 *C*. *butyricum* genomes [57,61]. Finally, each of the pangenome studies analysed the features of pathogenic *C. butyricum* strains [54,56]. Zou *et al*. [54] found that a total of 261 genes are unique to the BoNT/E-encoding strains and that the majority of these are involved in transcription and carbohydrate metabolism [54]. In addition to the BoNT/E genes, Pei *et al*. [55] used the Virulence Factor Database to identify a further 24 potential virulence factors across the genomes of pathogenic *C. butyricum* isolates [55]. Of these, the most frequently observed genes were associated with cell wall lipopolysaccharides and features of both the flagella and the bacterial capsule [55]. Finally, it was found that the 78 *C*. *butyricum* genomes analysed by Pei *et al*. [55] encode an average of 170 antibiotic resistance genes (ARGs) and that 55 of these are common to all strains [55]. Yang *et al*. [56] also determined that closely related strains share similar ARGs.

Herein, we describe the first comprehensive comparative analysis of the genome architecture of *C. butyricum*, using data from 16 completely assembled genomes. We identify a conserved ~0.8 Mb genetic element which satisfies the three core criteria of a chromid, and which is unique to *C. butyricum* amongst other *Clostridium* species. Using 162 additional genome assemblies, we also define several pathogen-specific genotypes, which include a set of genes involved in the biosynthesis of a novel CPS and genes required for the utilization of the mucin-derived monosaccharide, l-fucose.

## Methods

### Collection of *Clostridium* genomes

Twenty complete *C. butyricum* genome assemblies were retrieved from the NCBI genome database on 9 November 2023. Upon analysis, the genomes of strains SJ1, 29-1 and TK520 were removed due to an incomplete assembly, a high percentage of frameshifted proteins and a misassembled genome, respectively. A duplicate entry for strain DSM 10702 (NBRC 13949) was also excluded prior to analysis. At the time of submission (April 2025), we note that one additional complete genome assembly for the strain GBW-N1 has been deposited in the NCBI genome database. However, the genome size and organization of this strain do not deviate from those included in our analysis. A further 162 incompletely assembled *C. butyricum* genomes were downloaded from NCBI on 22 January 2025. These were refined from a total of 220 available *C. butyricum* genomes by removing duplicate entries, misassembled or chromosome-only assemblies, assemblies derived from metagenomes and assemblies with many frameshifted proteins. All available complete genome assemblies for the species *Clostridium beijerinckii*, *Clostridium felsineum*, *Clostridium saccharobutylicum* and *Clostridium saccharoperbutylacetonicum* were retrieved from NCBI on 9 November 2023, and the representative complete genomes of 35 further *Clostridium* species were downloaded from NCBI on 12 June 2024.

### Phylogenetic analyses

A phylogenetic tree was constructed using the complete genome sequences of 16 *C*. *butyricum* strains. The genomes were annotated using Prokka (prokka/1.14.5-gompi-2022b), and the output .gff files were aligned using Roary (Roary/3.13.0-foss-2022a), with the *-i* parameter set to 90 [62,63]. This revealed a total of 3,148 core genes, determined by their conserved presence across all strains. The IQ-TREE program (IQ-TREE/2.2.1-gompi-2021b) was then used to infer a maximum likelihood phylogenetic tree based on the core gene alignment, using 1,000 bootstrap replicates [64]. The resulting phylogenetic tree was visualized and edited using the Interactive Tree of Life tool (version 6.9) [65]. A further tree in which the genome of *C. saccharobutylicum* DSM 13864 was used as the outgroup was constructed using the same method.

The most closely related species to *C. butyricum* was determined by accessing the set of 92 core bacterial genes that was curated by Na *et al*. [66] to replace the use of 16S rRNA sequences in phylogenetic tree construction [66]. *C. butyricum* orthologues of genes from each of the nine Clusters of Orthologous Gene (COG) categories represented in the set were selected for BLASTp searches against the genomes of the type species of four closely related *Clostridium* species: *C. beijerinckii*, *C. felsineum*, *C. saccharobutylicum* and *C. saccharoperbutylacetonicum*. The Mauve multiple genome alignment software (version 2.4, progressiveMauve) was then used to align the sequences of the *C. butyricum* DSM 10702 chromid and the *C. saccharobutylicum* DSM 13864 chromosome [67].

### Bioinformatic analyses

The 669 genes of the DSM 10702 chromid were assigned to COG categories using eggNOG-mapper V2, which was run on Galaxy against the eggNOG 5.0.2 database [68]. A total of 82 genes without COG assignments, 125 genes of unknown function (COG category S) and 21 pseudogenes were excluded from the biological function analysis. Eleven genes were then reassigned to the mobilome category (COG category X), and eight genes were reassigned to an additional category (category Φ), determined by their predicted involvement in nitrogen metabolism. A total of 34 genes, which had been assigned to multiple COG categories, were assigned to a single category. The functions of genes present on the DSM 10702 chromid and the accessory plasmids of the strains CBM588, DSM 10702, JKY6D1 and TOA were predicted using the tools blast (Basic Local Alignment Search Tool), InterPro, RegPrecise and the CAZy database and guided by the automatic genome annotations assigned by Prokka, GenBank, RefSeq and the RAST Server [69,73].

Chromosomal and chromid-encoded core genes, determined by their conserved presence across all 16 complete *C. butyricum* genomes, were identified using the Roary gene presence/absence output. A total of 2,662 chromosomal and 486 chromid-encoded core genes were assigned to COG and Kyoto Encyclopedia of Genes and Genomes (KEGG) categories (as above), and the gene frequencies for each category were calculated as the number of genes present per megabase [68]. Gene functions that are enriched on the chromid were determined by calculating the percentage change in the gene frequency for each COG/KEGG category on the chromid compared to the chromosome. To test whether there is a statistical difference between the distribution of gene functions on the chromosome compared to the chromid, a chi-square (*χ*^2^) test for independence was carried out, using the null hypothesis that the proportion of genes in each COG/KEGG category is the same for the chromosome and the chromid. As the *χ*^2^ test requires at least 80% of the cells to have an expected count greater than five, only the 20 and 15 most populated COG and KEGG categories were included in the statistical analysis, respectively.

To identify genes which are unique to pathogenic strains, a total of 178 *C*. *butyricum* genomes at various stages of assembly were annotated using Prokka and aligned using Roary, to provide the gene presence/absence output. Filters were applied to the output table to display genes which are absent from all non-pathogenic strains, but present in pathogenic strains. The dRep program (version 2.0.0) was then used to group highly similar genomes [ANI (average nucleotide identity) >99.5%] and select a representative genome for each group [74]. Genes conserved in at least 3 of the 12 representative pathogenic strains were selected for further analysis.

### Identification of putative chromids in *Clostridium* species

The workflow described by diCenzo and Finan [75] and the strict chromid criteria set out by Harrison *et al*. [76] were used to identify putative chromids in *Clostridium* species [75,76]. On 12 June 2024, the NCBI genome database contained 336 *Clostridium* genomes of 40 *Clostridium* species. A representative genome and the associated metadata for each of these 40 *Clostridium* species were downloaded. First, genomes, which contained only one replicon, were eliminated. The largest replicon of each of the remaining genomes was then annotated as the chromosome, while the second largest replicon of each genome was highlighted for further assessment. For the latter, a minimum size cut-off of 0.25 Mb was used to shortlist potential chromids. Potential chromids were then distinguished from megaplasmids if their G+C content was within 2 mol% of that of the chromosome. Finally, these replicons were confirmed as chromids if they possessed a plasmid-type replication system and carried at least one core gene that is found on the chromosome in other species.

## Results

### *C. butyricum* has a conserved genome architecture

To understand the species diversity of *C. butyricum*, the type species of the genus *Clostridium*, a set of sequenced genomes along with other metadata were collected for analysis. A total of 16 high-quality complete genomes were used in the study, which include commensal strains isolated from human stool and animal intestines, strains prepared for use as probiotics, environmental isolates and pathogenic strains isolated from cases of food poisoning, infant botulism and NEC (Table 1) [20,37, 50, 77,85].

**Table 1.** *C. butyricum* genomes and metadata sourced from NCBI on 9 November 2023. Strains are ordered by total genome size from largest to smallest, and pathogenic strains are shown in bold

Strain	Isolation source	Total genome (Mb)	Chromosome (Mb)	Megaplasmid (Mb)	Plasmid(s) (bp)	GenBank accession	Reference
**CFSA3987**	NEC case, stool sample	4.75	3.86	0.88	–	GCA_009650315.1	[81]
**CFSA3989**	NEC outbreak, environmental swab	4.75	3.86	0.88	–	GCA_009650335.1	[81]
DSM 10702	Pig intestine	4.71	3.92	0.77	6,059, 8,060	GCA_014131795.1	[37]
**CFSA-TJ-E**	Botulism case, stool sample	4.7	3.95	0.75	–	GCA_024399875.1	[86]
**CDC_51208**	Botulism case	4.64	3.81	0.82	9,567	GCA_001886875.1	[79]
QXYZ514	Soil	4.64	3.87	0.77	–	GCA_026651935.1	[153]
4-1	Human stool sample	4.64	3.87	0.80	–	GCA_005145085.1	[80]
16-3	Human stool sample	4.63	3.87	0.77	–	GCA_013112415.1	[83]
KNU-L09	Human stool sample	4.63	3.82	0.80	–	GCA_001456065.2	[154]
DKU-11	Human stool sample	4.63	3.86	0.77	–	GCA_030389005.1	[78]
LV1	Shrimp intestine	4.63	3.86	0.77	–	GCA_027627495.1	[84]
JKY6D1	Pit mud	4.62	3.82	0.79	8,060	GCA_001465175.1	[50]
CBM588	Human stool sample	4.61	3.81	0.79	8,060	GCA_030758275.1	[85]
TOA	Probiotics	4.6	3.79	0.80	8,061	GCA_001646605.1	[20]
S-45-5	Human stool sample	4.59	3.81	0.78	–	GCA_003315755.1	[77]
CBUT	Probiotics	4.49	3.78	0.71	–	GCA_018140655.1	[82]

Across the 16 *C*. *butyricum* strains, the total genome sizes sit within a relatively narrow window of 4.49–4.75 Mb, and average 4.64±0.06 Mb (mean±sd). The chromosome, which ranges from 3.78 to 3.92 Mb, has an average size of 3.85±0.05 Mb and encodes between 4,050 and 4,309 genes, of which 3,852–4,112 are protein-coding. In addition to a ~3.9 Mb chromosome, a conserved feature of all 16 *C*. *butyricum* strains is the presence of a ~0.8 Mb megaplasmid. This carries between 625 and 782 protein-coding genes and ranges in size from 0.71 to 0.88 Mb, which accounts for between 16% and 19% of the total genome size. The mean size of the megaplasmid is 0.79±0.04 Mb, with a small interquartile range of 0.05 Mb. It is of note that the three largest megaplasmids, which range from 0.82 to 0.88 Mb, all belong to pathogenic strains (Table 1). We also note that the range of genome sizes observed for *C. butyricum* strains in our study is narrower than the 3.74–5.29 Mb range observed for *C. butyricum* isolates across the pangenome studies carried out by Zou *et al*. [54], Pei *et al*. [55] and Yang *et al*. [54,56]. However, we found that each of these studies has included several genome assemblies which have been annotated by NCBI as being either incomplete, contaminated or misassembled, so these values may not reflect the true range of genome sizes found across the species (Table S1, available in the online version of this article).

In addition to the megaplasmid, five strains possess smaller accessory plasmids. These are found in both pathogenic and non-pathogenic strains and range in size from 6,059 to 9,567 bp (Table 1). The only strain with multiple small accessory plasmids is DSM 10702. This strain carries the plasmids pCB_1 (6059 bp) and pCB_2 (8060 bp), which each encode nine genes. Although the role of most of these genes is unclear, it is predicted that pCB_1 encodes a zonula occludens toxin and a Rep protein. Likewise, pCB_2 is predicted to encode a bacteriocin, a Rep protein, a plasmid stabilization protein and the MobA and TraD conjugal transfer proteins, which suggests that the plasmid is transmissible. In support of this, BLASTn analyses showed that there is at least 99% nucleotide sequence identity between pCB_2 and each of the ~8 kb plasmids present in the strains JKY6D1, CBM588 and TOA, which suggests that this plasmid has been horizontally transmitted. Finally, the largest of the small accessory plasmids is pNPD4_1 of the botulism-associated strain CDC_51208. This 9,567 bp plasmid harbours eight genes, which are predicted to encode the conjugal transfer proteins MobA and TraD, a Hin recombinase, the plasmid replication protein ParB, a plasmid stabilization protein, a putative nitrite/sulphite reductase, a LuxR family protein and a DNA-binding protein of unknown function.

**Fig. 1.**
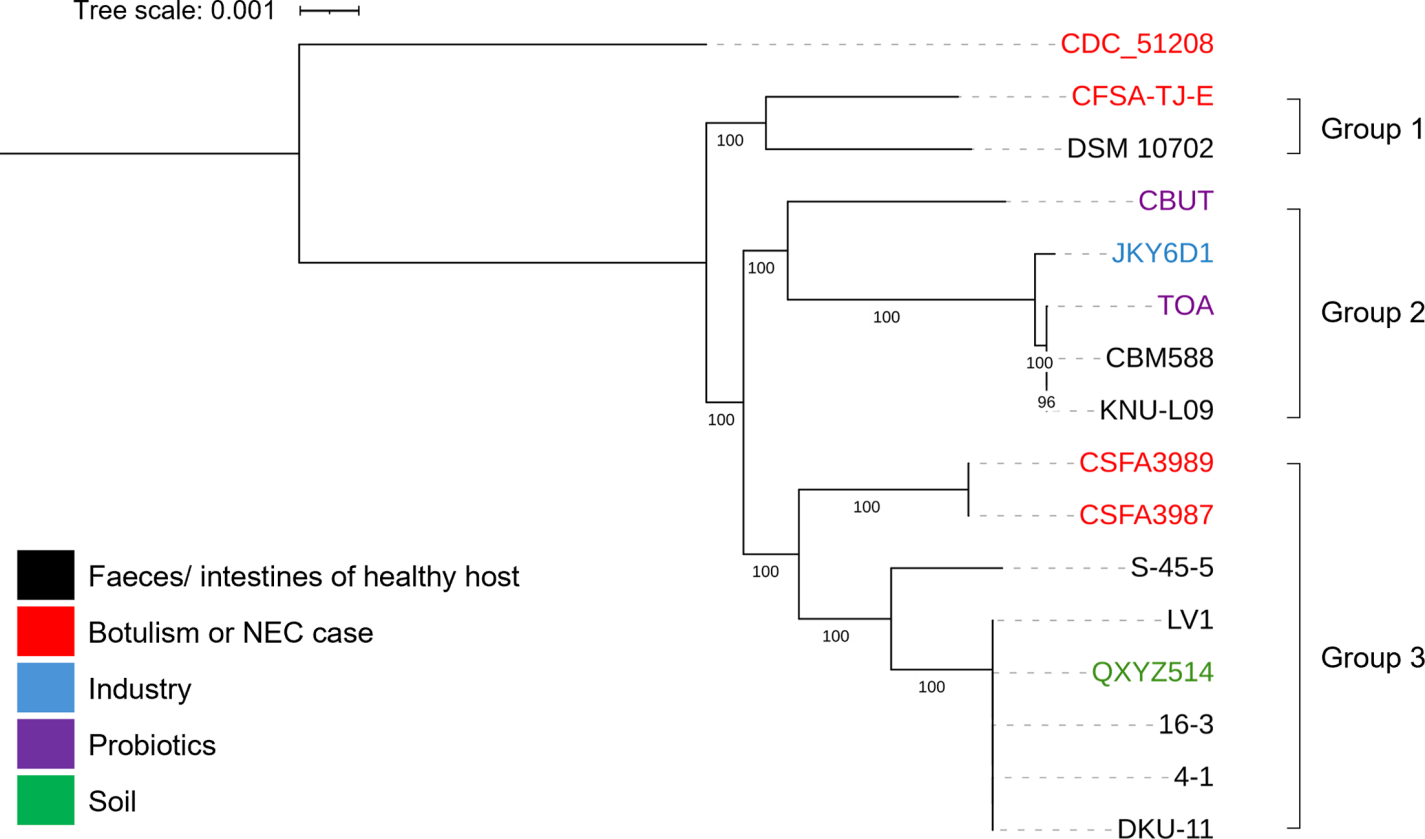
The phylogenetic relationships of the 16 *C. butyricum* isolates which have complete genome assemblies. The phylogenetic tree was constructed by a method of maximum likelihood based on the concatenation of 3,148 core genes. Numbers at the tree nodes represent bootstrap support values >90% (1,000 replications).

To further analyse the genomes of the 16 *C*. *butyricum* strains, a whole-genome single nucleotide polymorphism (SNP) tree was constructed (Fig. 1). Within this tree, three main groups can be observed (Fig. 1). Group 1 comprises the botulism-associated strain CFSA-TJ-E and the *C. butyricum* type strain, DSM 10702. Group 2 comprises the two probiotic strains (CBUT and TOA), a strain used in liquor fermentation (JKY6D1) and two commensal strains which were isolated from healthy human stool (CBM588 and KNU-L09). Group 3 comprises five further commensal strains (S-45-5, LV1, 16-3, 4-1 and DKU-11), both of the NEC-associated strains (CFSA3989 and CFSA3987) and a strain isolated from soil (QXYZ514). In addition to the three main groups, the botulism-associated strain CDC_51208 forms an additional, more distant branch (Fig. 1). Furthermore, the genomic difference observed between the two botulism-associated strains aligns with the two distinct known BoNT/E-producing *C. butyricum* clades, which produce either the toxin subtype BoNT/E4 (CDC_51208) or the subtype BoNT/E5 (CFSA-TJ-E) [79,86]. Aside from this, we note that there is no clear relationship between the source of each of 16 strains and their position on the tree and find that both pathogenic and non-pathogenic strains are found within Groups 1 and 3 (Fig. 1). However, a second phylogenetic tree, in which the close relative *C. saccharobutylicum* has been used as the outgroup, shows a tight grouping of the species *C. butyricum* (Fig. S1).

### A conserved chromid is a defining feature of the species *C. butyricum*

**Fig. 2.**
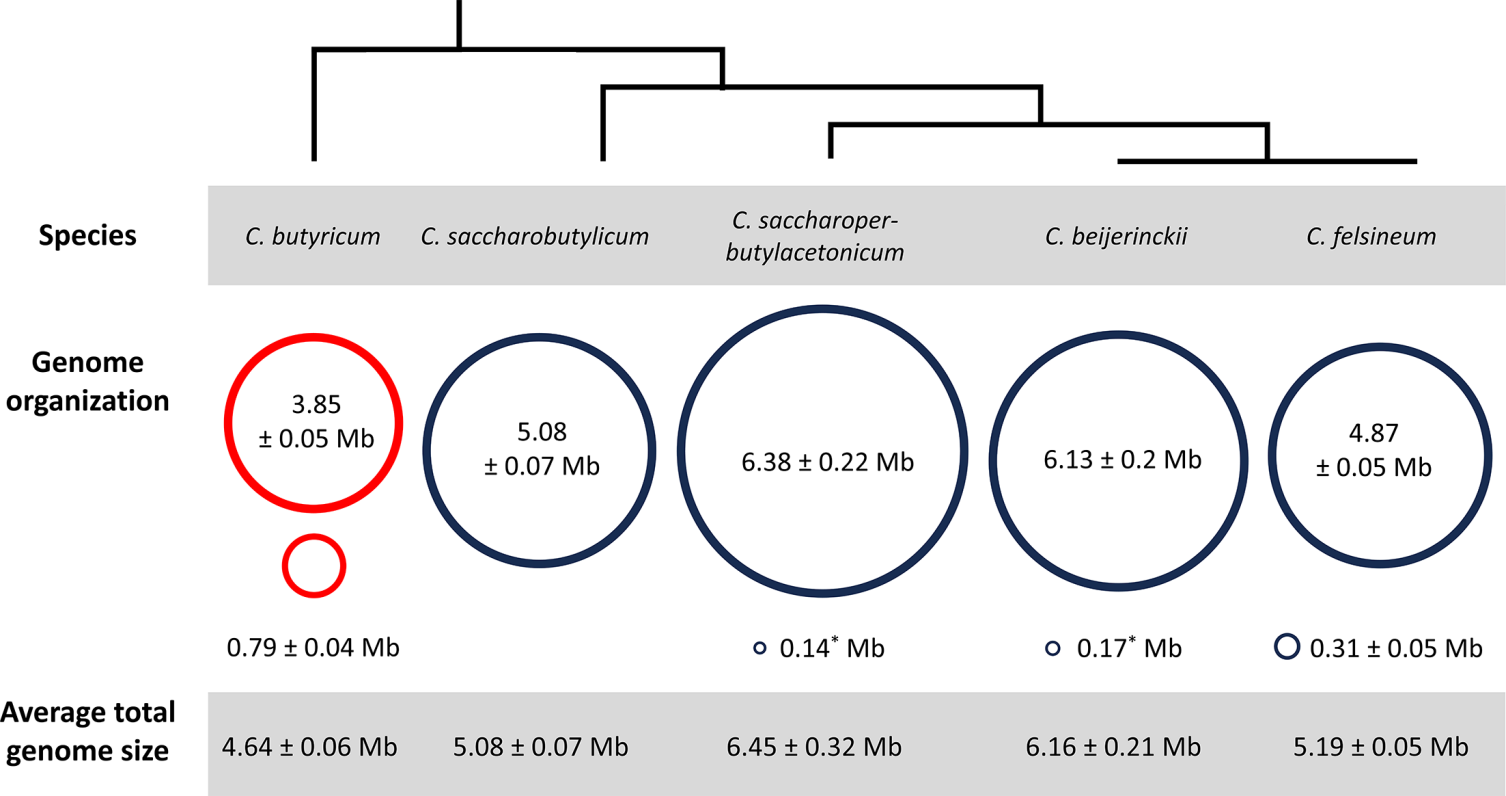
A visual summary of the genome architectures of *C. butyricum* and four related *Clostridium* species, showing the average sizes of the whole genome, chromosome and large second replicons (mean±sd). An approximate phylogenetic relationship between species is shown, as presented in Lawson and Rainey [1]. ^*^sd is less than 0.01.

A consistent feature of each of the 16 *C*. *butyricum* genomes is the presence of a ~0.8 Mb megaplasmid (Table 1). To establish whether the possession of a ~0.8 Mb genetic element and a ~3.9 Mb chromosome can be used to unambiguously distinguish *C. butyricum* from other *Clostridium* species, the genome architectures of several closely related *Clostridia* were analysed. These include *C. felsineum* (formerly *Clostridium roseum*), *C. beijerinckii*, *C. saccharobutylicum* and *C. saccharoperbutylacetonicum*, which form a clade with *C. butyricum* in the phylogenetic tree constructed by Lawson and Rainey [1] for the genus *Clostridium* (Fig. 2) [1]. The average genome sizes of *C. saccharobutylicum* (5.08±0.07 Mb), *C. felsineum* (5.19±0.05 Mb), *C. beijerinckii* (6.16±0.21 Mb) and *C. saccharoperbutylacetonicum* (6.45±0.32 Mb) are considerably larger than that of *C. butyricum* (4.64±0.06 Mb) (Fig. 2). The average size of the chromosome of these four species is also larger than the 3.85±0.05 Mb chromosome of *C. butyricum* (Fig. 2). It is clear that the possession of a ~0.8 Mb megaplasmid is unique to *C. butyricum* amongst closely related *Clostridium* species (Fig. 2).

Due to its large size, we considered whether this genetic element could be classified as a chromid, rather than a megaplasmid. Chromids are large bacterial extrachromosomal elements, which have three core defining criteria: a nucleotide composition close to that of the chromosome (usually within 1%), a plasmid-type maintenance and replication system and the possession of core genes that are found on the chromosome in other species [76]. To establish whether this conserved ~0.8 Mb replicon can be redesignated as a chromid, the features of the megaplasmid of the *C. butyricum* type strain, strain DSM 10702, were analysed.

**Fig. 3.**
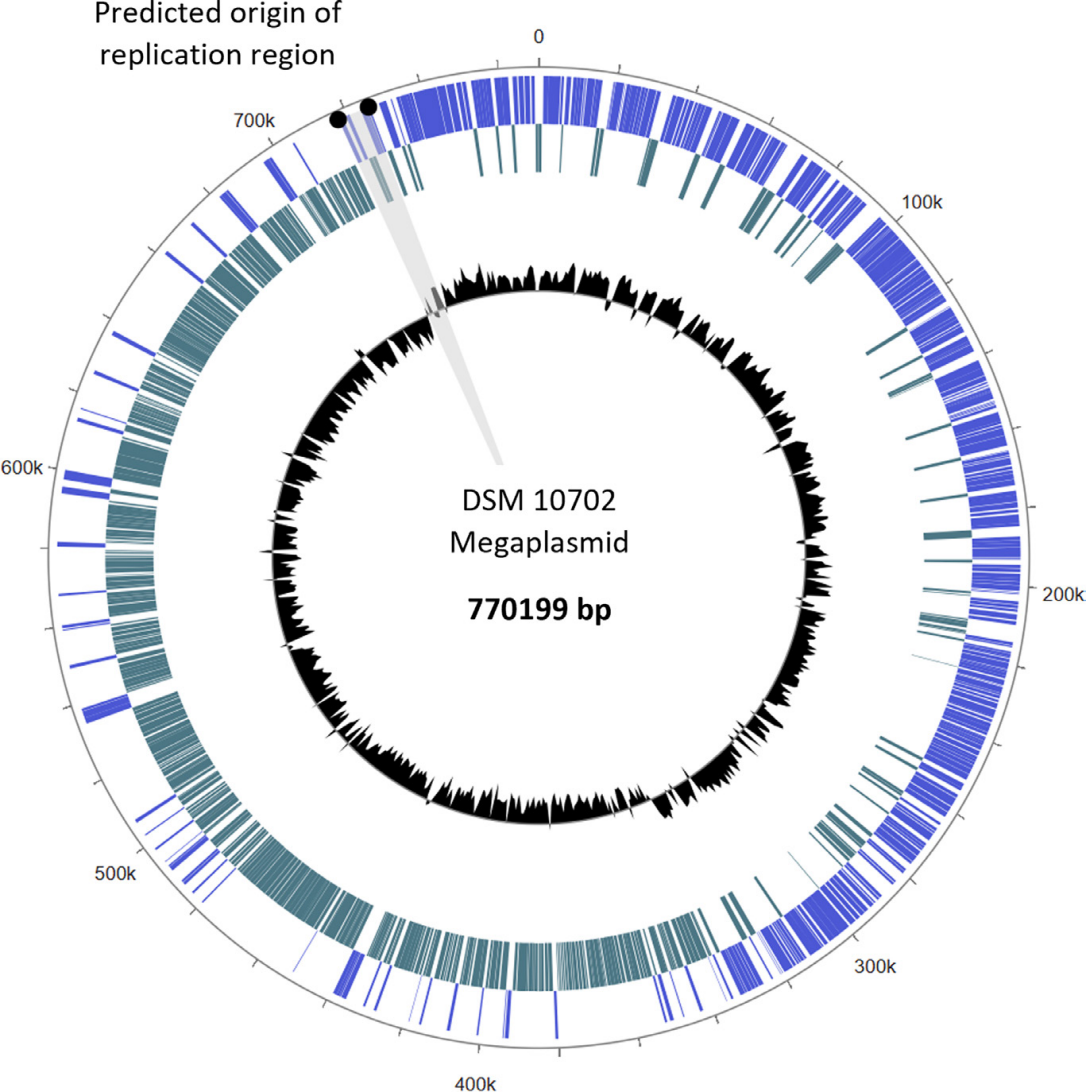
The megaplasmid of *C. butyricum* DSM 10702, indicating the region predicted to harbour the origin of replication. The inner track (black) presents the GC skew of the megaplasmid, and the two outer tracks represent genes encoded on the forward strand (blue) and reverse strand (teal). The megaplasmid was visualized using the MORF Genome Browser tool [87].

First, it was found that the G+C contents of the chromosome and megaplasmid are within 1 mol% of each other, at 28.8 and 28.3 mol%, respectively. This suggests that these two genetic elements have coexisted in the same cellular environment for a long period of time. Next, the DNA maintenance and replication systems of the megaplasmid were investigated. The Multi-Omics Research Factory (MORF) Genome Browser tool was used to visualize the switch in the GC skew of the megaplasmid, to predict the location of the origin of replication (Fig. 3) [87]. Within this region, genes which encode the putative plasmid replication proteins ParA, ParB and ParM were identified through homology to those present in other Gram-positive species. It was also found that site-specific tyrosine recombinases, which are involved in plasmid resolution, are encoded both directly upstream of *parM* and within the replication termination region of the megaplasmid [88].

Finally, for assessment against the third chromid criterion, we looked for megaplasmid-encoded core genes that are located on the chromosome in other *Clostridium* species. We examined which of the 669 genes encoded on the DSM 10702 megaplasmid have homologues in the bacterial minimal genome sets produced by Gil *et al*. and Ye *et al*. [89,90] (Table 2). This identified eight core genes, two of which encode ribose 5-phosphate isomerase A (RpiA) and dihydrofolate reductase (FolA), which are only present as single copies on the megaplasmid in *C. butyricum*, but are encoded on the main chromosome in related species (Table 2). The other six genes encode proteins with core functions in DNA replication and central metabolism, but the original copies of these genes are still present on the chromosome (Table 2). In addition to the DSM 10702 strain, it was found that the megaplasmids of the 15 other *C. butyricum* strains presented in Table 1 also satisfy the three core chromid criteria and encode the only genomic copy of the core gene *rpiA*. Henceforth, the ~0.8 Mb megaplasmid of *C. butyricum* can now be referred to as a chromid.

**Table 2.** Core genes that are located on the megaplasmid in *C. butyricum* DSM 10702 and their chromosomally encoded orthologues in other *Clostridium* species

Location of core gene	Category	Protein	*C. butyricum* DSM 10702		*C. acetobutylicum* ATCC 824	*C. beijerinckii* NCIMB 8052	*C.* *saccharoperbutylacetonicum* N14(HMT)
			Megaplasmid	Chromosome	Chromosome	Chromosome	Chromosome
Megaplasmid only	Biosynthesis of cofactors	Dihydrofolate reductase (FolA)	FF104_20005	*	CA_C1495	Cbei_2234	Cspa_c22880
	Pentose phosphate pathway	Ribose 5-phosphate isomerase (RpiA)	FF104_20920	*	CA_C1431	Cbei_0761, Cbei_2367	Cspa_c30010, Cspa_c34030
Copies on both the megaplasmid and the chromosome	Basic replication machinery	DNA polymerase III subunit alpha (PolC)	FF104_20855	FF104_05715†	CA_C3442	Cbei_3203	Cspa_c30050
	Glycolysis	Phospho-pyruvate hydratase	FF104_18260	FF104_14880†	CA_C0713	Cbei_0602	Cspa_c43930, Cspa_c51060
		Fructose-6-phosphate aldolase	FF104_18035, FF104_20820	FF104_03350†	CA_C1347	Cbei_2386, Cbei_4454, Cbei_4645	Cspa_c23570, Cspa_c43440, Cspa_c46740, Cspa_c46910
	Pentose phosphate pathway	Transketolase	FF104_18040, FF104_20815	FF104_03355†	CA_C0944, CA_C1348	Cbei_0532, Cbei_2387, Cbei_4453, Cbei_4644	Cspa_c23580, Cspa_c46720, Cspa_c46850

* Note that non-homologous genes which encode enzymes with the same predicted functions are also encoded on the chromosome. The chromosomal homologue of RpiA is encoded by the gene *rpiB* (FF104_15590), and the chromosomal homologue of FolA is encoded by *DHFR* (FF104_00815). However, the major form of each isoenzyme is encoded by the gene present on the megaplasmid.

†Chromosomal copies of the megaplasmid-encoded core genes.

Analysis of a further set of 39 *Clostridium* genomes showed that none contained a non-chromosomal genetic element that fulfils all the criteria for a chromid (Tables S2 and S3, available in the online Supplementary Material). This allows us to conclude that the possession of a chromid is not a typical feature of the genus *Clostridium*, despite being a core feature of the type species, *C. butyricum*.

### Biological functions of chromid-encoded genes

As the ~0.8 Mb chromid is a hallmark of a *C. butyricum* genome, we wished to examine more deeply which genes it contains, beyond those defined as ‘core’ genes by others. To give an initial overview of its function, the 669 genes present on the chromid of strain DSM 10702 were assigned to COG categories (Table S4).

After transcription (COG category K), the largest proportion (17%) of genes belong to the carbohydrate transport and metabolism category (COG category G). Those which are unique to the chromid include a complete set of genes for the uptake and degradation of trehalose and xylosides, as well as glycosidases involved in the breakdown of arabinogalactan, fucosides and glucosides. However, an example of a ‘split’ metabolic pathway is seen with the *N-*acetylglucosamine (GlcNAc) catabolic pathway, as two putative NagA enzymes are encoded on the chromosome, while the single NagB homologue is encoded on the chromid (Table S4 and Fig. S2). This differs from the location of these genes in the genomes of the closely related species *C. saccharobutylicum* and *C. beijerinckii*, in which NagA and NagB are encoded together on the chromosome. A second example is the d-xylose catabolic pathway in which the first two enzymes of the pathway, namely XylA-II and XylB, are only encoded on the chromid, while the d-xylose transporter is encoded on the chromosome (Table S4 and Fig. S2). Moreover, Scalfaro *et al*. [53] demonstrate that the loss of the large second replicon from two pathogenic *C. butyricum* strains results in the inability to grow on d-xylose, which directly supports the hypothesis that important growth-related genes are encoded on the chromid [53].

In addition to carrying genes for the metabolism of additional carbohydrates, the chromid carries genes for the utilization of ethanolamine as an alternative nitrogen source, as well as the complete set of *nif* genes, which likely confer the potential for nitrogen fixation (Tables 3 and S4). Moreover, it was found that the organization of the *nif* genes is largely conserved in *C. acetobutylicum* and *C. beijerinckii* but that these genes are encoded on the chromosome in these species (Table 3).

**Table 3.** The complete set of *nif* genes encoded on the chromid in *C. butyricum* DSM 10702 and their chromosomally encoded orthologues in the species *C. acetobutylicum* and *C. beijerinckii*

Product	*C. butyricum* DSM 10702	*C. acetobutylicum* ATCC 824	*C. beijerinckii* NCIMB 8052
NifH, nitrogenase II	FF104_18300	CAC0253 (*nifH*)	Cbei_1999 (*nifH*) Cbei_0623 (*nifH*)
Nitrogen regulatory protein P-II	FF104_18305	CAC0254 (*nifHD*)	Cbei_2000 (*nifD*)
Nitrogen regulatory protein P-II	FF104_18310	CAC0255 (*nifHD*)	Cbei_2001 (*nifD*)
NifD, nitrogenase MoFe protein subunit alpha	FF104_18315	CAC0256 (*nifD*)	Cbei_2002 (*nifD*)
NifK, nitrogenase MoFe protein subunit beta	FF104_18320	CAC0257 (*nifK*)	Cbei_2003 (*nifK*)
NifE, nitrogenase MoFe cofactor biosynthesis protein	FF104_18325	CAC0258 (*nifE*)	Cbei_2004 (*nifE*)
NifN-B, nitrogenase cofactor biosynthesis protein	FF104_18330	CAC0259 (*nifN-B*)	Cbei_2005 (*nifB*) Cbei_0620 (*nifN-B*) Cbei_0621 (*nifN-B*) Cbei_0631 (*nifN-B*) Cbei_0632 (*nifN-B*)
NifVω, homocitrate synthase subunit omega	FF104_18340	CAC0260 (*nifVω*)	Cbei_2011 (*nifVω*)
NifVα, homocitrate synthase subunit alpha	FF104_18345	CAC0261 (*nifVα*)	Cbei_2012 (*nifVα*)

The energy production and conversion category (COG category C) also makes up a significant proportion (10%) of the chromid-encoded genes. Importantly, these include the only genomic copies of genes that encode glycerol dehydratase and 1,3-propanediol dehydrogenase, which are responsible for the production of the industrially relevant fermentation product 1,3-propanediol, as well as the enzyme pyruvate-formate lyase which is required for the production of formate, another of the main fermentation products of *C. butyricum* [91,94] (Table S4 and Fig. S2).

The remaining chromid-encoded genes are involved in a wide range of biological functions including defence mechanisms and the uptake and metabolism of inorganic ions, coenzymes, amino acids and nucleotides. Genes which encode transporters for inorganic ions (COG category P), which are often required for coenzyme biosynthesis (COG category H), account for 10% of the chromid-encoded genes. As well as additional transporters for the uptake of iron, magnesium, potassium and zinc, the chromid encodes the only genomic copies of transporters required for the uptake of molybdate and cobalt (Table S4). As expected, the two functional molybdate transporters are located directly upstream of the iron-molybdenum nitrogenase of the *nif* operon. However, all except one of the genes required for the biosynthesis of the cobalt-requiring coenzyme cobalamin are exclusively present on the chromosome, rather than alongside the chromid-encoded cobalt transporters [95]. Furthermore, the chromid encodes the only genomic copy of a gene required for the biosynthesis of pantothenate, a key precursor for coenzyme A biosynthesis (Fig. S2). Although it has been established that *C. butyricum* DSM 10702 is prototrophic for amino acids, the chromid encodes transporters for the uptake of serine/threonine, branched-chain amino acids and dipeptides, in addition to the only genomic copy of a gene required for tryptophan biosynthesis [96] (Table S4). Finally, it was noted that over half of the genes within the defence mechanisms category (COG category V) are unique to the chromid. These are clustered in a region of the chromid and encode two *β*-lactamases and several multidrug efflux pumps (Table S4).

**Fig. 4.**
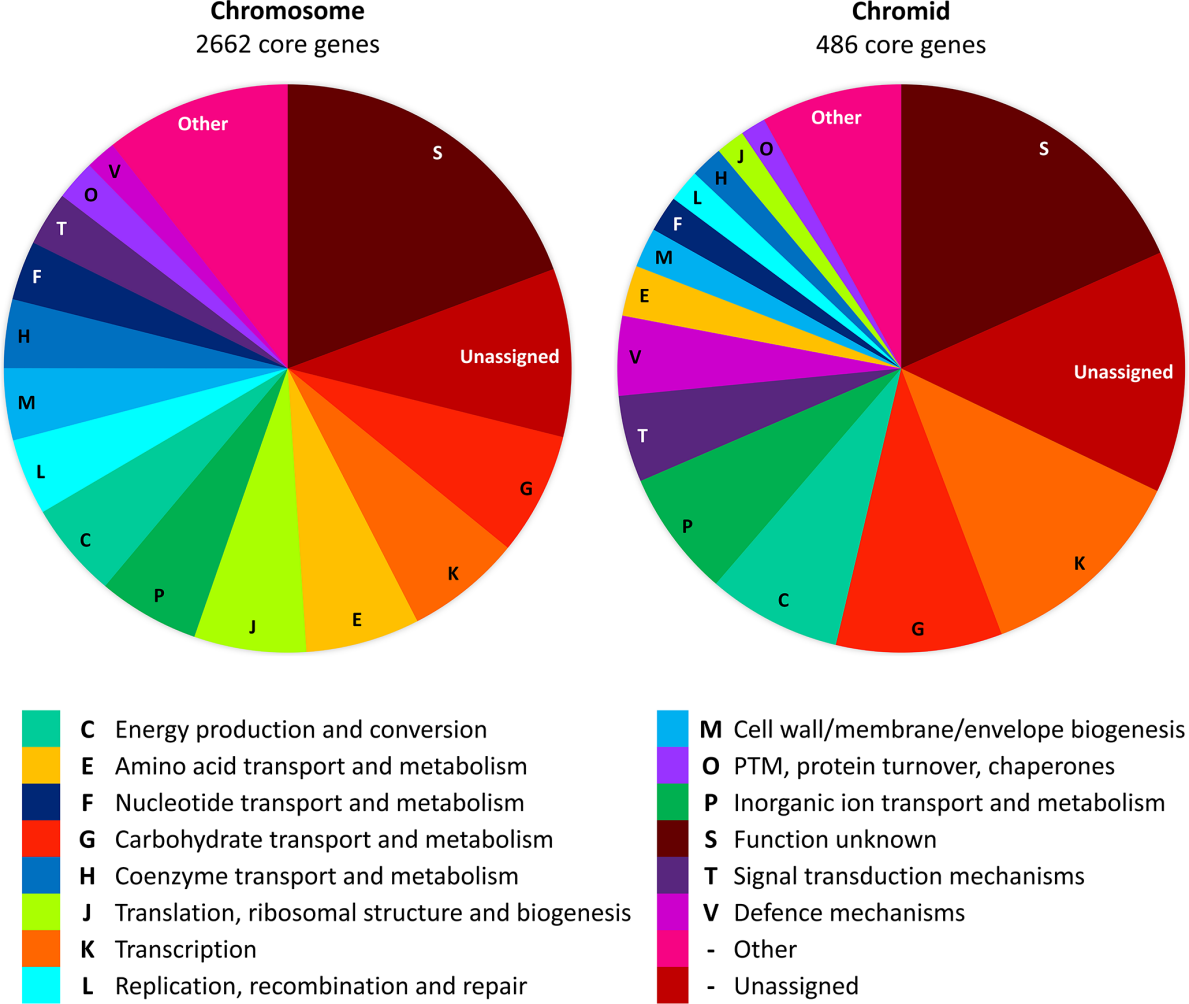
The composition of core genes on the chromosome compared to the chromid, determined by their COG category functions. The ‘other’ category includes all COG categories which contain less than 1% of genes on the chromosome or chromid.

To further explore the role and potential origin of the chromid, the functions of conserved genes on the chromid and chromosome were compared. Of the total pool of 5,844 chromosomal and 1,411 chromid-encoded genes, 46 and 34% represent conserved genes, determined by their presence in all 16 *C*. *butyricum* strains. These were assigned to COG and KEGG functional categories to assess their roles (Tables S5 and S6). The two most populated COG categories on both replicons are transcription (COG category K) and carbohydrate transport and metabolism (COG category G), which account for a larger proportion of the conserved genes encoded on the chromid than the chromosome (21 and 14%, respectively) (Fig. 4 and Table S5). There is also an enrichment of genes on the chromid for 15 further COG categories and five KEGG categories, which include those involved in xenobiotic degradation, signal transduction and the transport and metabolism of inorganic ions (Fig. 4, Tables S5 and S6). On the other hand, genes involved in core processes such as translation, replication and cell division represent a higher proportion of core genes on the chromosome than the chromid (Fig. 4). Moreover, it was found that there is a significant difference between the gene frequencies observed for the COG and KEGG categories on the chromid versus the chromosome: *χ*^2^ (19, *N*=851)=85.88, *P*=0.01 (COG) and *χ*^2^ (14, *N*=310)=36.42, *P*=0.01 (KEGG). Both the enrichment of genes involved in accessory functions and the lower frequency of genes involved in core functions on the chromid compared to the chromosome suggest that the chromid may have formed from an ancient *C. butyricum* megaplasmid via the acquisition of several essential genes.

### Pathogenic *C. butyricum* strains encode conserved virulence factors

The whole-genome SNP tree constructed for the 16 complete *C. butyricum* genomes shows that the four pathogenic strains are not monophyletic and that virulence has evolved from multiple evolutionary ancestors within the species (Fig. 1). To attempt to elucidate the genetic features involved in pathogenicity and host colonization, we assembled a larger group of strains by accessing a further 162 incompletely assembled *C. butyricum* genomes, which include 12 additional botulism isolates and 45 additional NEC isolates (Tables 4 and S7). Highly genetically similar strains (ANI >99.5%) were grouped, and a representative genome was selected for each set, to provide a total of four representative botulism isolates and eight representative NEC isolates (Table 4).

**Table 4.** The predicted roles and distribution of the six pathogen-associated gene clusters across the 12 representative pathogenic *C. butyricum* strains isolated from cases of botulism and NEC. Ticks indicate the presence of the cluster

Representative Strain		Predicted function					
		BoNT/E complex	CPS biosynthesis	Flagellar glycosylation	Agr system	Copper resistance	Drug efflux
**Botulism -associated strains**	**BoNT E BL5262**	✓	✓	✓	✓	✓	✓
	**CDC_51208**	✓	✓	✓	✓	✓	✓
	**LCL-155**	✓				✓	✓
	**CFSA-TJ-E**	✓					
**NEC-associated strains**	**NOR 33234**		✓	✓			
	**374**		✓				
	**359**				✓		
	**376**						
	**353**						
	**365**						
	**372**						
	**CFSA3987**						

We find that overall, botulism isolates appear genetically distinct from NEC isolates. Of the 2,605 pathogen-specific genes that were identified, 1,726 (66%) are exclusively present in the genomes of at least one NEC isolate, while 739 (28%) are exclusively found in the genomes of at least one botulism isolate (Fig. S3). Although we were unable to identify any pathogen-specific genes that are conserved across the genomes of all pathogenic strains, we find that a total of 105 genes, which are mainly located within six discrete clusters across the genome, are each conserved in at least 3 of the 12 representative pathogenic strain backgrounds (Table S8 and Fig. S3). Moreover, three of these clusters are present in both botulism and NEC isolates, which suggests that these encode common traits for immune evasion or enhanced colonization, which are not directly related to the pathology of the two different disease types (Table 4).

**Fig. 5.**
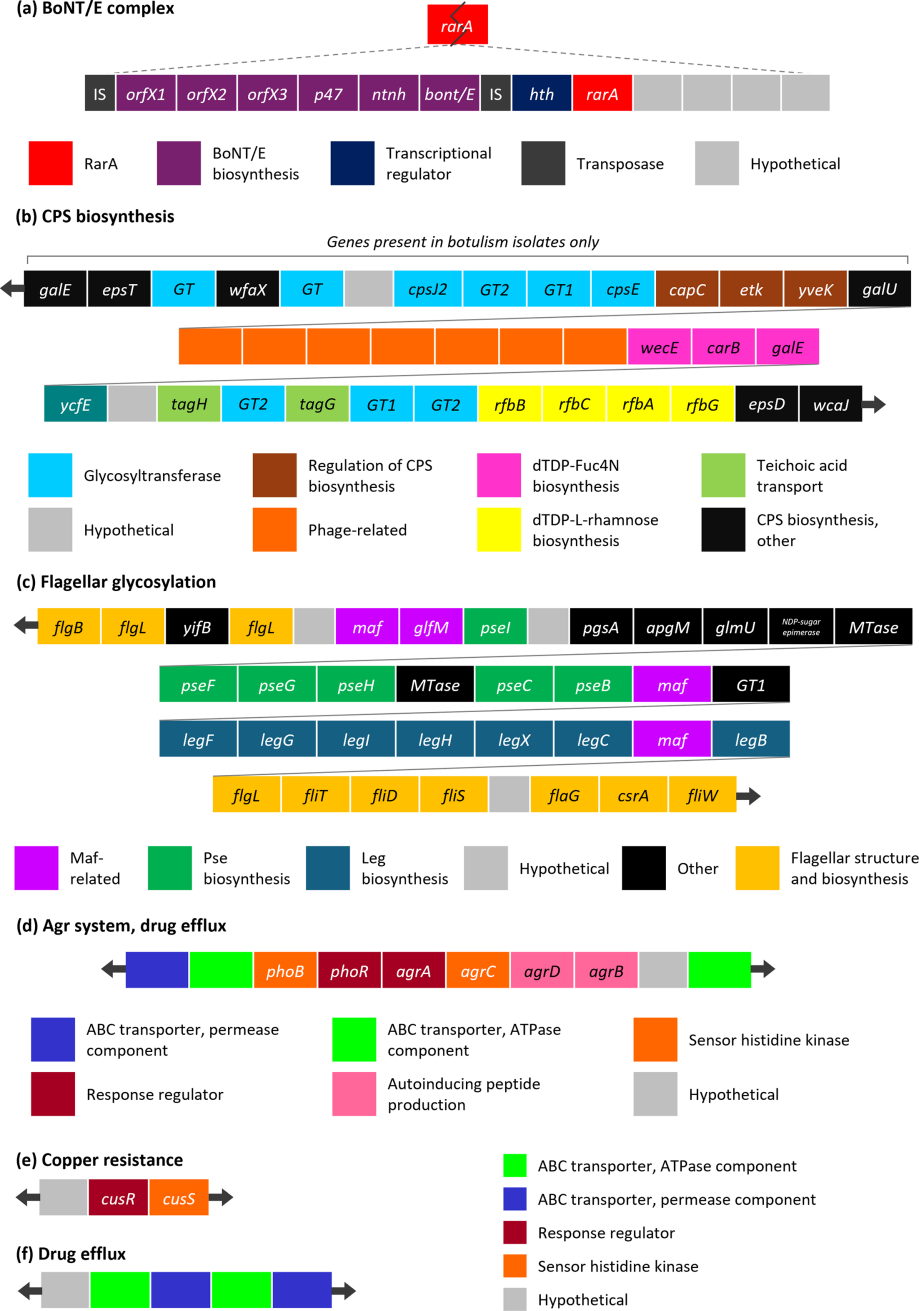
Gene clusters which are conserved in several pathogenic *C. butyricum* strains. (**a**) Genes which encode the components of the BoNT/E complex. (**b**) Genes predicted to be involved in the biosynthesis of a CPS. (**c**) Genes predicted to be involved in flagellar glycosylation with nonulosonic acids. (**d**) Genes of the Agr system and those involved in drug efflux. Genes involved in (**e**) resistance to copper ions and (**f**) drug efflux.

As expected, we find that the 12-gene cluster which encompasses the six genes of the botulinum neurotoxin E (BoNT/E) operon is conserved in all botulism-associated strains (Tables 4, S8 and Fig. 5a). The BoNT/E operon comprises *bont/e* and five genes whose products are predicted to associate with BoNT/E to provide protection and enhancement of toxicity (*orfX1*, *orfX2*, *orfX3*, *p47* and *ntnh*) [97]. Encoded downstream of *bont/e* is a helix-turn-helix transcriptional regulator, an intact copy of the *rarA* gene and four hypothetical proteins, two of which are predicted to be involved in recombination (Fig. 5a). Furthermore, it was noted that the cluster is present in the genome as an insertion in a split *rarA* gene (Fig. 5a). In two of the four representative botulism isolates, the 5′ portion of the split *rarA* gene is located directly upstream of the BoNT/E operon, while in the remaining two strains, three conserved transposase sequences separate the 5′ partial *rarA* gene from *orfX1*. Comparative genome analysis with related *Clostridium* species revealed that this cluster was also likely acquired from *C. botulinum* via horizontal gene transfer.

The first of the three gene clusters that can be found in both NEC and botulism isolates has a predicted role in the biosynthesis of a CPS (Table 4). This cluster comprises ten genes, that encode dTDP-4-amino-4,6-dideoxy-d-galactose (dTDP-Fuc4N) biosynthetic genes, three glycosyltransferases and two genes that are homologous to TagH and TagG, the ATP-binding and permease subunits of a teichoic acid transporter, respectively (Fig. 5b and Table S8). In botulism-associated strains only, a further 14 conserved genes, which have homology to the CPS biosynthesis genes of the probiotic bacterium *Lactiplantibacillus plantarum*, are encoded directly upstream of the nine phage-related genes which precede the conserved CPS cluster (Fig. 5b). These include glycosyltransferases responsible for adding rhamnose, galactose and glucose monomers to a CPS; a flippase for transporting undecaprenyl pyrophosphate-linked units across the membrane; three tyrosine kinases which are involved in the regulation of CPS biosynthesis [98]. Finally, in both NEC and botulism-associated strains, a further six CPS-related genes are encoded downstream of the conserved cluster (Fig. 5b). These are predicted to encode dTDP-l-rhamnose biosynthesis genes, a membrane-bound O-antigen family polymerase and an enzyme involved in the transfer of UDP-galactose onto a lipid carrier. A comparison of the genes in this region to those in related *Clostridium* species revealed that they have likely been acquired from *C. botulinum* via horizontal gene transfer. The 16 genes downstream of the conserved phage cluster are also conserved in the pathogenic species *Clostridium neonatale*, and orthologues of genes from across the entire CPS locus are encoded together in the genome of the pathogenic anaerobe *Fusobacterium pseudoperiodonticum*. Overall, it is likely that the genes in this region are involved in the biosynthesis of a CPS which incorporates galactose, rhamnose, glucose, GlcNAc, Fuc4N and a teichoic acid-like moiety.

The second of the three common clusters contains 37 genes and spans part of the flagellar biosynthesis locus (Fig. 5c). Encoded within this cluster are flagellar structural proteins, proteins involved in flagellar assembly and enzymes with predicted roles in the biosynthesis and modification of nonulosonic acids (Fig. 5c and Table S8). The presence of nonulosonic acid biosynthesis genes within the flagellar locus suggests an involvement in flagellar glycosylation, which has been well documented for the gastrointestinal pathogens *Helicobacter pylori* and *Campylobacter jejuni* [99,102]. The final cluster of genes that is shared by both botulism and NEC isolates contains ten genes, which are predicted to encode the permease and ATPase components of a macrolide ABC (ATP-binding cassette) efflux transporter, the ATPase component of a multidrug ABC efflux transporter, the PhoB-PhoR two-component regulatory system and homologues of the four genes of the *agrACDB* locus (Fig. 5d and Table S8). In several other pathogenic species, which include *Escherichia coli* and *Vibrio cholerae*, it has been shown that the PhoR/PhoB two-component regulatory system is involved in gut colonization and bacterial virulence, through the regulation of biofilm formation and the expression of cell surface components, via links with quorum-sensing circuits [103,106]. Likewise, it has been established that the *agrACDB* quorum-sensing system coordinates the global regulation of virulence genes in *Staphylococcus aureus* [107,108]. Within this system, AgrD and AgrB are responsible for producing an autoinducing peptide, which binds and activates the sensor histidine kinase AgrC, resulting in phosphorylation of the response regulator, AgrA [107]. In addition to this conserved *agrACDB* locus, it was found that other accessory gene regulator (Agr) system components are present across both pathogenic and non-pathogenic *C. butyricum* strains. These include an AgrB homologue, which is present in all but two of the 178 strains included in the analysis, up to two additional AgrB homologues and up to three AgrA homologues. However, it was noted that in pathogenic strains that contain the conserved *agrACDB* locus, only one AgrA homologue is encoded in the genome.

The final two conserved pathogen-associated clusters are exclusively present in botulism-associated strains and are predicted to have roles in antimicrobial resistance (Table 4). The first of these encodes homologues of a copper-responsive two-component system, CusRS, and is likely involved in tolerance to high levels of copper ions (Fig. 5e). The second cluster encodes two ABC-type multidrug efflux transporters and a putative secreted lipoprotein, which is conserved in the gastrointestinal pathogens *Clostridioides difficile* and *C. neonatale* (Fig. 5f).

### A catabolic pathway for host-derived fucose is characteristic of pathogenic strains of *C. butyricum*

**Fig. 6.**
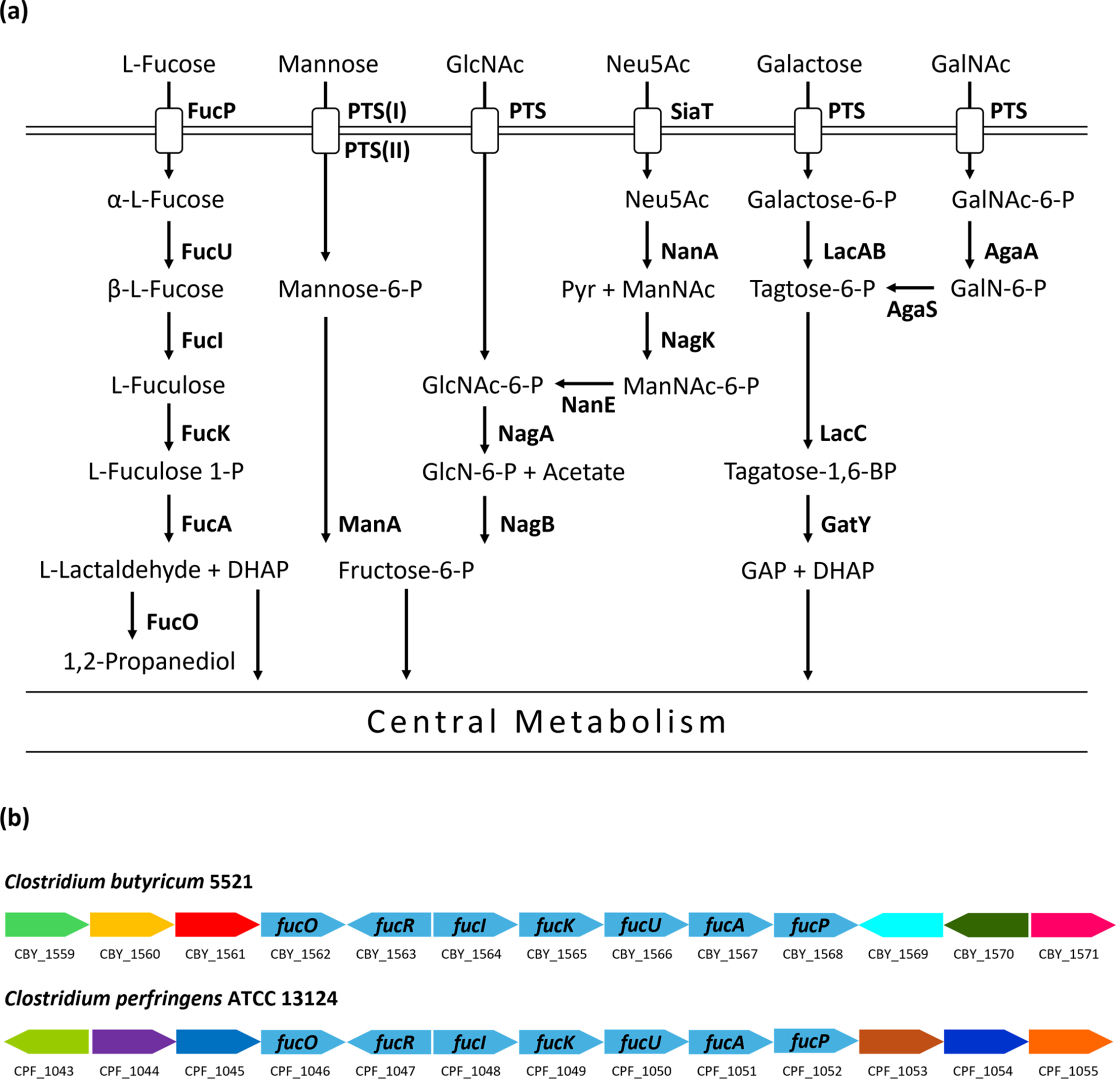
(**a**) The predicted uptake routes and catabolic pathways for the six major monosaccharides present in host colonic mucin glycans. Pathways were determined using known orthologues in other *Clostridia* and Gram-positive mucin-degrading bacteria. FucP, l-fucose-proton symporter; FucU, l-fucose mutarotase; FucI, l-fucose isomerase; FucK, l-fuculokinase; FucA, l-fuculose phosphate aldolase; FucO, lactaldehyde reductase; NagA, *N*-acetylglucosamine-6-phosphate deacetylase; NagB, glucosamine-6-phosphate deaminase; SiaT, sialic acid transporter; NanA, *N*-acetylneuraminate lyase; NagK, *N*-acetylglucosamine kinase; NanE, *N*-acetylmannosamine-6-phosphate 2-epimerase; LacAB, galactose-6-phosphate isomerase; LacC, tagatose-6-phosphate kinase; GatY, tagatose-1,6-bisphosphate aldolase; AgaA, *N*-acetylgalactosamine-6-phosphate deacetylase; AgaS, galactosamine-6-phosphate isomerase; ManA, mannose-6-phosphate isomerase. (**b**) The organization of genes in the fucose catabolic gene clusters present in *C. butyricum* in 5521 and *C. perfringens* ATCC 13124. The organization of these genes is identical across the *C. butyricum* strains 5521, CDC_51205, BoNT E BL6262 and BL-5262-9RE.

In addition to the aforementioned virulence factors, it was hypothesized that in order to compete with the resident microbiota, pathogenic *C. butyricum* strains may possess additional catabolic capabilities for mucin-derived sugars compared to non-pathogenic strains. A survey was therefore carried out to determine the distribution of genes involved in the catabolism of the six main monosaccharides present in the glycans of host colonic mucin, across five pathogenic and five non-pathogenic * C. butyricum* strains, which were isolated from a range of sources (Fig. 6a and Table 5) [109,111].

**Table 5.** The predicted presence of complete uptake and catabolic pathways for the six major monosaccharides present in host mucin glycans, across five pathogenic (bold) and five non-pathogenic *C. butyricum* strains. Ticks indicate the presence of a complete uptake and catabolic pathway, and blank cells indicate an absent or incomplete pathway

Strain	Isolation source	Monosaccharide degradation pathway					
		l-Fucose	GlcNAc	Neu5Ac	Galactose	GalNAc	Mannose
**CSFA3987**	NEC case		✓*	✓*		✓	✓*
**CDC_51208**	Botulism case	✓	✓*	✓*	✓	✓	✓*
**5521**	Botulism case	✓	✓*	✓*	✓	✓	✓*
**BoNT E BL5262**	Botulism case	✓	✓*	✓*	✓	✓	✓*
**BL-5262-9RE**	Unknown	✓	✓*	✓*	✓	✓	✓*
DSM 10702	Pig intestine		✓*	✓*	✓	✓	✓*
LV1	Shrimp intestine		✓*	✓*	✓	✓	✓*
JKY6D1	Pit mud		✓*	✓*	✓	✓	✓*
TOA	Probiotics		✓*	✓*	✓	✓	✓*
4-1	Human stool sample		✓*	✓*	✓	✓	✓*

*Pathways whose components are encoded across both the chromosome and the chromid.

The analysis revealed that all ten strains possess a complete set of genes for the uptake and catabolism of *N*-acetylgalactosamine (GalNAc), GlcNAc, mannose and sialic acid (Neu5Ac) (Tables 5 and S9). Furthermore, each strain encodes a complete galactose uptake and catabolic pathway, except for the NEC-associated strain CFSA3987, in which the three galactose phosphotransferase system transporter subunits are absent (Tables 5 and S9). However, it was found that four out of the five pathogenic strains encode a complete l-fucose degradation pathway, while l-fucose catabolic genes are absent from all non-pathogenic strains (Tables 5 and S9). Further analysis of the l-fucose catabolic gene cluster revealed that it is highly conserved in several related pathogenic species, which include *C. perfringens* and *Clostridium baratii* (Fig. 6b and Table S10).

## Discussion

*C. butyricum*, the type species of the genus *Clostridium*, is both a taxonomically important organism and a species with considerable biological diversity. The completion of closed genomes, which capture some of this diversity, has provided an opportunity to study fundamental features of the genome organization of this species. The first and most significant structural feature that we identified is a ~0.8 Mb chromid, which is conserved in the genome of all sequenced *C. butyricum* strains and which we argue is a unique characteristic of this species. To date, the only report of the presence of a chromid in a *Clostridium* species is in the genome of *Clostridium bornimense* M2/40^T^, which was isolated from a biogas reactor [112]. However, this 0.7 Mb replicon does not carry core genes and thus should not be formally described as a chromid (Table S3). Moreover, our finding that the possession of a chromid is unique to *C. butyricum* amongst other *Clostridium* species is supported by the work of diCenzo and Finan [75] [75]. By categorizing the replicons from 8,302 complete bacterial genomes, which included two *C. butyricum* strains (JKY6D1 and KNU-L09) and 106 strains from 28 other *Clostridium* species, it was found that *C. butyricum* is the only *Clostridium* species which possesses putative chromids [75]. Furthermore, Franciosa *et al*. [51] analysed the genomes of ten neurotoxigenic *C. butyricum* strains using pulsed-field gel electrophoresis and found that each contained a large megaplasmid with an estimated size of >610 to ~825 kb, in line with our discovery of a ~0.8 Mb chromid in sequenced *C. butyricum* strains [51]. However, our finding that the chromid is confined to a single species within the genus *Clostridium* differs from the observations made by Harrison *et al*. [76] that the possession of a chromid is a genus-specific character, with chromids found in all sequenced representatives of several genera [76]. Nevertheless, it is of note that all 82 of the chromid-possessing genomes included in this initial analysis belong to Gram-negative phyla [76].

To support our classification of the second replicon of *C. butyricum* as a chromid, we identify eight chromid-encoded genes that belong to the bacterial minimal genome sets curated by Gil *et al*. [89] and Ye *et al*. [90], and which are encoded on the chromosome in other species [89,90]. Although these account for only 1.2% of the total genes encoded on the chromid, this value supports the findings of Harrison *et al*. [76] that the majority of core genes are present on the chromosome [76]. Furthermore, in other chromid-containing species, it has been found that core genes account for a similarly low proportion of between 0.1 and 3.6% of the total chromid-encoded genes [113,116]. For each of the core genes on the *C. butyricum* chromid, we determine that copies or non-orthologous alternatives are also present on the chromosome, which suggests that the chromid may be dispensable. However, by the definition set out by Harrison *et al*. [76], core genes include those which become essential ‘either under all conditions or environmentally’ [76]. The carriage of genes which may become indispensable under certain conditions is likely, considering the conservation of this replicon across the species, despite the high metabolic burden associated with the maintenance of a large second replicon [117]. For example, we find that the gene which encodes the tryptophan synthase alpha subunit is present on the chromid only, while the beta subunit is encoded on both the chromid and the chromosome. Therefore, although a tryptophan transporter is encoded on the chromosome, the chromid would become essential for tryptophan biosynthesis under tryptophan-limiting conditions. The hypothesis that the chromid becomes essential under certain environmental conditions is also supported by the work of Scalfaro *et al*. [53], which demonstrated that the large second replicons of two pathogenic *C. butyricum* strains are essential for survival at 15 °C and for tolerance to low pH and high salinity [53]. The authors also show that the ability to metabolize 5 out of a panel of 14 carbon sources is lost in strains which have been cured of their megaplasmids. This supports our finding that several genes that are required for the catabolism of sugars such as xylose and ribose are only present on the chromid [53]. Likewise, we find that the chromid carries the only genomic copies of several genes required for the utilization of more complex carbon sources such as arabinogalactan and fucosides, which may be required when more simple sugars are scarce. Genes required for the utilization of several mucin-derived sugars, which are in abundant supply in the colon, are also encoded across both the chromid and the chromosome, which highlights the importance of the chromid for gut-dwelling *C. butyricum* strains. Scalfaro *et al*. [53] also demonstrate that the growth rates of *C. butyricum* strains which have been cured of their megaplasmids are significantly lower than that of their WT parental strains [53]. In line with this, we identify several chromid-encoded genes which, despite not being present in the minimal bacterial gene set, are conserved in at least 95% of *C. butyricum* genomes and are likely to be important for viability [54,56]. These include genes involved in butyrate fermentation, nitrogen fixation and the metabolism of alginate and trehalose, which are all located solely on the chromid [54,56].

In addition to imparting the ability to utilize additional carbon sources, we suggest that the possession of a chromid provides several other fitness benefits in *C. butyricum*. First, we show that the chromid is enriched in genes involved in defence mechanisms, which include several antibiotic efflux pumps and antibiotic resistance proteins. These likely aid survival in the densely populated gut environment, where bacteria are exposed to antimicrobials produced by members of the gut microbiome. We also find that the chromid encodes the only genomic copies of several transporters required for the uptake of amino acids, cofactors and nucleotides. The ability to take up these organic compounds when they are abundant in the environment is advantageous, due to the greater energetic cost of their *de novo* synthesis. Furthermore, the possession of a chromid may facilitate an increased rate of bacterial growth, as has been observed in chromid-bearing species of the genera *Rhizobium* and *Sinorhizobium* [118]. It has been suggested that dividing the genome in this way allows for the possession of a smaller chromosome and for both replicons to be replicated concurrently, thus decreasing the time taken to replicate the genome [119,120]. In line with this, we find that the chromosome of *C. butyricum* is smaller than that of closely related *Clostridium* species (~4.64 and ∼5.26 Mb, respectively) and that the doubling times observed for *C. butyricum* strains are less than those of its closest relatives [121,127].

Overall, we suggest that the low frequency of essential genes and the functional bias of genes on the chromid compared to the chromosome reflect the origin of the chromid as an ancient *C. butyricum* megaplasmid (the ‘plasmid hypothesis’), rather than from a schism of an ancestral *C. butyricum* chromosome (the ‘schism hypothesis’), which would result in a relatively equal distribution of essential genes between the two replicons [76,128, 129]. Our finding that the distribution of conserved gene functions on the chromosome differs from that of the chromid is supported by the global COG analysis carried out by diCenzo and Finan [75], which determined that chromosomes are enriched in core functions, while chromids, which likely evolved from megaplasmids, are enriched in genes involved in transcription, carbohydrate transport and metabolism, inorganic ion transport and metabolism and signal transduction (COG categories K, G, P and T, respectively) [75]. To further disprove the schism hypothesis for the formation of the chromid, we examined the genome of the most closely related species to *C. butyricum*, namely *C. saccharobutylicum* (Table S11). We determined that orthologues of genes found on the chromid of *C. butyricum* are widely dispersed across the chromosome of *C. saccharobutylicum*, rather than clustered in a region of the chromosome that subsequently split away to form the chromid (Fig. S4).

In the second part of our study, we identify pathogen-specific genes which are likely to be involved in immune evasion and colonization of the intestine. A major source of nutrients in the colon are the heavily glycosylated mucin proteins that form the main component of the intestinal mucus layer [130]. Members of the gut microbiome have adapted to utilize the monosaccharides present in these glycans, which make up ~80% of the total mucin biomass [131]. To allow establishment in the gut, some pathogenic species have acquired genes for the utilization of mucin-derived monosaccharides, which are cleaved from mucin glycans by commensal bacteria [132,133]. We find that genes required for the catabolism of l-fucose, which is predominantly present at the terminal position of mucin glycans, are exclusively present in pathogenic *C. butyricum* strains and were likely acquired from *C. baratii* via horizontal gene transfer [134]. It is predicted that these genes confer a competitive advantage for colonization of the gut, as has been demonstrated for the gut pathogens *C. jejuni*, *Klebsiella pneumoniae* and *Salmonella* Typhimurium [133,135,137].

Although previous studies have suggested that haemolysin-like proteins and neuraminidases contribute to the virulence of NEC strains, we do not identify these as unique features of pathogenic *C. butyricum* isolates and find that sialic acid catabolic genes are present across diverse *C. butyricum* genomes [46,48]. However, we identify three novel gene clusters that are conserved in both NEC and botulism isolates, which include those predicted to be involved in the biosynthesis of a novel CPS. Previously, CPS compositions have only been described for strains of one *Clostridium* species, *C. perfringens* [138,142]. These are serotypically distinct and incorporate a wide variety of monomers [138,142]. Moreover, it was determined that a CPS of *C. perfringens* ATCC 13124, which is composed of a repeating trisaccharide unit comprised of rhamnose, galactose and GalNAc, has a role as a bacteriophage receptor [141,142]. We predict that the CPS produced by pathogenic *C. butyricum* strains also incorporates rhamnose and galactose subunits, in addition to glucose, GlcNAc and the amino sugar Fuc4N. Rhamnose-rich CPSs have also been identified in several other gut pathogens, which include *Enterococcus faecalis* and *Streptococcus mutans*, where they have roles in host colonization, antimicrobial resistance and protection from environmental stresses [143,145]. Within the CPS cluster of pathogenic *C. butyricum* strains, we also identify the two subunits of an ABC transporter that is homologous to the TagGH teichoic acid exporter of *Bacillus subtilis* [146]. As the teichoic acid biosynthetic genes are located elsewhere in the genome, it is hypothesized that teichoic acid-like moieties are used to decorate the CPS backbone in these pathogenic strains. This has previously been observed in the species *E. faecalis*, in which teichoic acid side chains present on a rhamnose-rich CPS were found to contribute to pathogenicity [147]. Overall, it is likely that the CPS produced by pathogenic *C. butyricum* strains plays a role in host colonization and survival within the gut environment.

In both botulism and NEC isolates, we also identify homologues of the *agrACDB* quorum-sensing locus of *S. aureus*, which has an established role in the global regulation of virulence genes [107,108]. One and two Agr-like quorum-sensing systems have also been identified in the genomes of the pathogenic species *C. perfringens* and *C. botulinum*, respectively [148,151]. These have been shown to be involved in sporulation, biofilm production and the regulation of extracellular toxin production [148,151]. We therefore predict that the *agrACBD* locus present in pathogenic *C. butyricum* strains also has a role in the regulation of virulence gene expression.

Overall, we suggest that virulent *C. butyricum* strains have evolved multiple times from related non-pathogenic ancestors via the acquisition of pathogen-specific genes, which is reflected by the recent findings of Chapman *et al*. [152]. In this study, the authors recognize that, in addition to the well-studied role of *C. perfringens* as a gastrointestinal pathogen, several *C. perfringens* strains, which lack the perfringolysin O toxin gene, function in promoting neonatal gut health [152]. This parallels our study, by highlighting the genetic and hence phenotypic diversity within a single species.

In summary, for the first time, we analyse the genome architecture of the species *C. butyricum*. We identify a conserved 0.8 Mb chromid, which carries genes that are predicted to become essential under certain environmental conditions. Although * C. butyricum* is the type species of *Clostridium*, we find that the possession of a chromid is unique to *C. butyricum* amongst other members of the genus and can therefore be used to distinguish *C. butyricum* strains from other *Clostridium* species. We also determine the genetic basis of novel virulence factors present in pathogenic *C. butyricum* strains, which include the l-fucose catabolic genes and genes which are predicted to encode a novel CPS. Further investigation of the structure and function of this CPS may help to expand our understanding of the way in which *C. butyricum* strains cause NEC.

## Supplementary material

10.1099/mgen.0.001477 Uncited Supplementary Material 1.

## Abbreviations

ABC: ATP-binding cassette
Agr: accessory gene regulator
ANI: average nucleotide identity
ARGs: antibiotic resistance genes
blast: Basic Local Alignment Search Tool
BoNT/E: botulinum neurotoxin type E
COG: Clusters of Orthologous Gene
CPS: capsular polysaccharide
dTDP-Fuc4N: dTDP-4-amino-4,6-dideoxy-D-galactose
GalNAc: *N*-acetylgalactosamine
GlcNAc: *N*-acetylglucosamine
KEGG: Kyoto Encyclopedia of Genes and Genomes
MORF: Multi-Omics Research Factory
NCBI: National Center for Biotechnology Information
NEC: necrotizing enterocolitis
SCFAs: short-chain fatty acids
SNP: single nucleotide polymorphism
*χ* ^2^: chi-square
