# Supplementary material for: Comparative genomics of Clostridium butyricum reveals a conserved genome architecture and novel virulence-related gene clusters
Source: Microb Genom. 2025 Aug 12;11(8):001477. doi: 10.1099/mgen.0.001477 (PMC12342839; doi:10.1099/mgen.0.001477)
Supplement: Uncited Supplementary Material 1. [file mgen-11-01477-s001.pdf]

Supplementary Material

**Table S1**	....	2
**Fig. S1**	....	3
**Table S2**	....	4
**Table S3**	....	5
**Table S4**	....	7
**Fig. S2**	....	14
**Table S5**	....	15
**Table S6**	....	16
**Table S7**	....	19
**Fig. S3**	....	22
**Table S8**	....	24
**Table S9**	....	26
**Table S10**	....	27
**Table S11**	....	28
**Fig. S4**	....	29
**References**	....	30

Table S1. *C. butyricum* strains which have been included in the pangenome studies of Zou et al. (2021), Pei et al. (2023) and Yang et al. (2024), but which have been annotated by NCBI as having errors in their genome assemblies (9–11).

Strain	GenBank Accession	Reference	NCBI Description of Error(s)	Zou et al., 2021	Pei et al., 2023	Yang et al., 2024
29-1	GCA_005145065.2	-	Many frameshifted proteins	✓	✓	✓
360	GCA_963668865.1	(1)	Contaminated	n/a†	n/a†	✓
B067	GCA_947381565.1	(2)	Incomplete	n/a†	n/a†	✓
BSD3178071175st1_B11_BSD3178071175_160912	GCA_040714095.1	-	Contaminated	n/a†	n/a†	✓
DS501	GCA_017352175.1	-	Chromosome only	n/a†	n/a†	✓
GGCC_0151	GCA_017565995.1	(3)	Incomplete	n/a†	n/a†	✓
INCQS635	GCA_000765285.1	(4)	Incomplete; many frameshifted proteins	*	✓	✓
MALS002	GCA_030035785.1	(5)	Incomplete	n/a†	n/a†	✓
NEC8	GCA_001458815.2	(6)	Incomplete; many frameshifted proteins	✓	✓	
S3	GCA_009735365.2	(7)	Incomplete	n/a†	n/a†	✓
SJ1	GCA_030291175.1	-	Incomplete	n/a†	n/a†	✓
SU1	GCA_001411625.1	-	Incomplete; many frameshifted proteins	✓	✓	
TK520	GCA_001746555.1	-	Misassembled	*	✓	✓
UBCB70	GCA_003935945.1	(8)	Contaminated	✓	✓	✓

*Authors have actively excluded the genome assembly from their pangenome analysis. †Assembly not available in the NCBI genome database at the time the respective pangenome study was carried out.

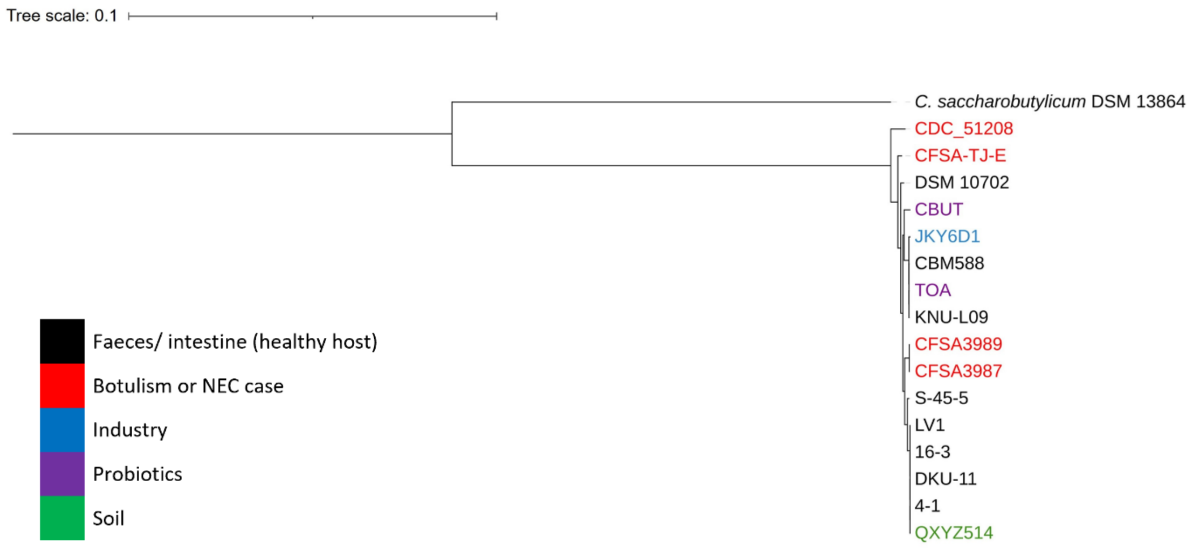

Fig S1. The phylogenetic relationships of the 16 *C. butyricum* isolates which have complete genome assemblies, using the strain *C. saccharobutylicum* DSM 13864 as the outgroup. The phylogenetic tree was constructed by a method of maximum likelihood.

Table S2. Genome data and GenBank accession numbers for *C. saccharobutylicum*, *C. beijerinckii*, *C. felsineum* and *C. saccharoperbutylacetonicum*, and the assessment of the megaplasmids present in these species against the three core chromid criteria. Cells are shaded grey for strains in which the megaplasmid is smaller than 0.25 Mb minimum size threshold for a chromid. The genomes were downloaded from NCBI on 9th November 2023.

Species	Strain	GenBank Accession	Reference	Genome (Mb)	Chromosome (Mb)	Megaplasmid (Mb)	Chromid Assessment†		
							G+G Content Difference*	Plasmid-Type Replication System	Core Genes
C. saccharobutylicum	DSM 13864	GCA_000473995.1	(12)	5.11	5.11	-			
	BAS/B3/SW/136	GCA_002003325.1	(13)	5.11	5.11	-			
	NCP 195	GCA_002003385.1	(13)	5.11	5.11	-			
	NCP 200	GCA_002003285.1	(13)	5.11	5.11	-			
	NCP 258	GCA_002003365.1	(13)	4.95	4.95	-			
C. felsineum	DSM 794	GCA_002006355.2	-	5.24	4.89	0.34	0	Yes	No
	DSM 7320	GCA_002006215.2	-	5.21	4.87	0.33	0	Yes	No
	DSM 793	GCA_002006235.2	-	5.17	4.92	0.24			
	DSM 6424	GCA_002006175.2	-	5.13	4.8	0.33	0	Yes	No
C. beijerinckii	NCIMB 14988	GCA_000833105.2	-	6.49	6.49	-			
	CloBei18h	GCA_025962575.1	-	6.46	6.29	0.17			
	DSM 6423	GCA_900010805.1	-	6.41	6.38	-			
	NRRL B-598	GCA_000506785.4	(14)	6.19	6.19	-			
	BAS/B3/I/124	GCA_002003345.1	-	6.12	6.12	-			
	CBEI	GCA_018140595.1	(15)	6.01	6.01	-			
	NCIMB 8052	GCA_000016965.1	(16)	6.00	6.00	-			
	SA-1	GCA_000767745.1	(17)	6.00	6.00	-			
	ASCUSDY20	GCA_021729445.1	-	5.98	5.98	-			
	DSM 791	GCA_018223745.1	(18)	5.95	5.88	-			
C. saccharoper-butylacetonicum	N1-4(HMT)	GCA_000340885.1	(19)	6.67	6.53	0.14			
	N1-504	GCA_002003305.1	-	6.22	6.22	-			

*G+C content difference between the megaplasmid and its associated chromosome. †We examined the other, smaller megaplasmids present in the genomes of closely related *Clostridium* species. Only three megaplasmids, which belong to three of the four *C. felsineum* genomes, are larger than the 0.25 Mb size cut-off which was previously defined for replicons with a G+C content less than 40% (20). These have an average size of 0.33 ± 0.005 Mb, harbour a plasmid-type replication system and have a G+C content within 1% of the chromosome. However, these replicons do not encode core genes and thus cannot be designated as chromids.

Table S3. Genetic information for the 23 *Clostridium* species whose genomes contain more than one replicon. Genomes were downloaded from NCBI on 12th June 2024. Strains have been ordered by the size of their second largest replicon, from largest to smallest. Cells have been shaded grey for species where the second replicon is smaller than 0.25 Mb minimum size threshold for a chromid.

***Clostridium* Species**	**Reference Strain, GenBank Accession and Reference**	**Total Genome (bp)**	**Number of Replicons**	**Chromosome (bp)**	**Second Largest Replicon (bp)**	**G+C Content Difference**	**Plasmid-Type Replication System**	**Core Genes**
C. butyricum	CDC_51208 GCA_001886875.1 (21)	4639914	3	3809831	820516	-1	Yes	Yes
C. bornimense	M2/40 GCA_000577895.1 (22)	3617025	2	2917864	699161	-2*	Yes	No
C. felsineum	DSM 794 GCA_002006355.2	5242194	3	4885251	337217	0	Yes	No
C. acetobutylicum	DSM 1731 GCA_000218855.1 (23)	4145581	3	3942462	191996	0		
C. estertheticum	CF001 GCA_030585465.1	4942908	3	4678732	191693	-1		
C. tagluense	CM022 GCA_026650945.1	5450588	4	5029099	182841	-1		
C. taeniosporum	1/k GCA_001735765.2 (24)	3503506	4	3264813	163055	-1		
C. argentinense	89G GCA_002074155.1	4803058	2	4662988	140070	-2.5		
C. saccharoper-butylacetonicum	N1-4(HMT) GCA_000340885.1	6666445	2	6530257	136188	0		
C. novyi	150557 GCA_003614235.1 (25)	2499078	3	2296219	134627	-1.5		
C. baratii	CDC51267 GCA_001991075.2	3211717	2	3091050	120667	-3		
C. tetani	Massachusetts substr. E88 GCA_000007625.1 (26)	2873333	2	2799251	74082	-4		
C. beijerinckii	DSM 791 GCA_018223745.1 (18)	5950247	2	5876902	73345	-2		
C. tyrobutyricum	KCTC 5387 GCA_001642655.1 (27)	3134437	2	3071606	62831	-3.5		
C. kluyveri	NBRC 12016 GCA_000010265.1	3955303	2	3896121	59182	1.5		

C. carboxidivorans	P7 GCA_001038625.1 (28)	5752782	2	5732880	19902	-2.5	
C. botulinum	ATCC 3502 GCA_000063585.1 (29)	3903260	2	3886916	16344	-1	
C. perfringens	CPI 18-6 GCA_020138775.1 (30)	3275424	2	3260088	15336	-2.5	
C. gasigenes	CGAS001 GCA_017348895.1	4132257	6	4110498	10796	-2	
C. aceticum	DSM 1496 GCA_001042715.1 (31)	4207037	2	4201318	5719	-4	
C. septicum	DSM 7534 GCA_003606265.1	3404718	2	3399422	5296	-3.5	
C. cadaveris	IFB3C5 GCA_020911725.1 (32)	3625784	3	3619347	4819	-3	
C. chauvoei	12S0467 GCA_002327205.1 (33)	2889569	2	2885628	3941	-1.5	

*Although this replicon has a G+C content of 28.09%, which is within 2% of that of the chromosome (29.87%), this is greater than the <1% difference typically observed between chromids and their associated chromosome (20).

Table S4. The COG categories, predicted functions and locus tags of genes which are only encoded on the DSM 10702 chromid (bold), in addition to chromid encoded genes for which gene copies or genes with the same predicted function are also encoded on the chromosome.

COG Category	Subcategory	Protein	Locus Tag
C	Energy production and conversion	**Glycerol dehydratase**	FF104_18420
		**Ferredoxin--NADP reductase**	FF104_18440
		**Pyruvate-formate lyase**	FF104_18555
		**Nitroreductase**	FF104_18695
		**NAD-dependent dihydropyrimidine dehydrogenase subunit PreA**	FF104_18985
		**FprA family A-type flavoprotein**	FF104_19100
		**Hypothetical protein**	FF104_19555
		**1,3-Propanediol dehydrogenase**	FF104_19590
		**Molybdopterin cofactor-binding domain-containing protein**	FF104_19880
		**Xanthine dehydrogenase family protein subunit M**	FF104_19885<br>FF104_19890
		**Dihydroorotate dehydrogenase**	FF104_19900<br>FF104_20515
		**FAD-binding oxidoreductase**	FF104_20050
		**Epoxyqueuosine reductase**	FF104_20380
		**Superoxide dismutase**	FF104_20500
		**Molybdopterin oxidoreductase**	FF104_20895
		**Desulfoferrodoxin**	FF104_20980
		**Aldo/keto reductase, uncharacterised**	FF104_21180
		**FAD-binding oxidoreductase**	FF104_21245
		Nitroreductase	FF104_17930
		FAD-dependent oxidoreductase	FF104_18225
		Glycerol dehydrogenase	FF104_18380<br>FF104_20360<br>FF104_19625
		Pyruvate formate lyase-activating protein	FF104_18425<br>FF104_18660<br>FF104_18560
		Glycerol-1-phosphate dehydrogenase	FF104_18430<br>FF104_20800
		Formate C-acetyltransferase	FF104_18655
		Hydroxyacid dehydrogenase	FF104_19295
		Hydroxylamine reductase	FF104_19360
		D-isomer specific 2-hydroxyacid dehydrogenase	FF104_19395
		Sulfite reductase	FF104_19630
		Xanthine dehydrogenase	FF104_19895
		Flavodoxin	FF104_20550
		FAD-dependent oxidoreductase	FF104_20600
		L-lactate dehydrogenase	FF104_20835
		Sulfite reductase subunit C	FF104_20935
		Anaerobic sulfite reductase subunit AsrB	FF104_20940
		Rubrerythrin	FF104_20985
		Ferredoxin--NADP reductase	FF104_21025
D	Cell division	**Plasmid partition protein ParA**	FF104_21095
		Transglutaminase domain-containing protein	FF104_19415
E	Amino acid metabolism	**GNAT family N-acetyltransferase**	FF104_18160
		**Homocitrate synthase**	FF104_18340<br>FF104_18345
		**Allantoate amidohydrolase**	FF104_18955<br>FF104_20795
		**Tryptophan synthase subunit alpha**	FF104_19000
		**DegT/DnrJ/EryC1/StrS aminotransferase family protein**	FF104_19170
		**M14 carboxypeptidase**	FF104_19185
		**M20 peptidase beta-alanine synthase**	FF104_19285<br>FF104_19300
		**Arginine utilisation protein**	FF104_19390
		**Arginase**	FF104_19695
		**Peptidase M20**	FF104_19925
		**Diaminopropionate ammonia-lyase**	FF104_19930
		**Glutathionylspermidine synthase**	FF104_20155
		**5-Methyltetrahydropteroyltriglutamate-homocysteine S-methyltransferase**	FF104_21000

		Tryptophan synthase subunit beta	FF104_18995
		DegT/DnrJ/EryC1/StrS aminotransferase family protein	FF104_19180
		VOC family protein	FF104_19325
	Amino acid transport	**EamA family transporter**	FF104_17980<br>FF104_19610
		**MFS transporter**	FF104_18355<br>FF104_20125
		**Branched-chain amino acid permease**	FF104_18820
		**Serine/ threonine transporter SstT**	FF104_21175
		ABC-type dipeptide/oligopeptide/nickel transport system, periplasmic binding protein	FF104_18835
		ABC-type dipeptide/oligopeptide/nickel transport system, ATPase component	FF104_18850
**F**	Nucleotide transport	**Nucleobase:cation transporter**	FF104_19310
		Purine permease	FF104_19440
		Uracil permease	FF104_21240
	Nucleotide metabolism	**Dihydropyrimidinase**	FF104_18980<br>FF104_19910
		**Membrane bound hypothetical protein**	FF104_19040
		**Adenosine deaminase**	FF104_19215
		**Nucleotide deaminase**	FF104_19905
		**Guanine deaminase**	FF104_19935
		**Nucleoside phosphohydrolase**	FF104_20040
		Anaerobic ribonucleoside-triphosphate reductase	FF104_18055
		Phosphoribosylaminoimidazolecarboxamideformyltransferase	FF104_18155
		Adenylate kinase	FF104_20015
**G**	*Mono-/di-/tri-saccharide transport and metabolism*		
	Mannose transport	**Mannose PTS transporter (I)**	FF104_17945<br>FF104_17950<br>FF104_17955
		**Mannose PTS transporter (II)**	FF104_19800<br>FF104_19805<br>FF104_19790<br>FF104_19795
	Ribose transport and metabolism	**D-ribose pyranase**	FF104_18015
		**Ribose ABC transporter**	FF104_18025<br>FF104_18030
		**Ribose 5-phosphate isomerase A**	FF104_20920
		Ribose-5-phosphate isomerase B	FF104_19155
	Neu5Ac/ GlcNAc transport and metabolism	**NagC transcriptional regulator**	FF104_18075<br>FF104_20825
		**Glucosamine-6-phosphate deaminase**	FF104_19050
		ABC-transporter, GlcNAc	FF104_19465
	Sucrose metabolism	Sucrose-6-phosphate hydrolase	FF104_18370
	Glycerol transport and metabolism	Dihydroxyacetone kinase subunit DhaK	FF104_18385
		Glycerol ABC transporter	FF104_18085<br>FF104_18090<br>FF104_18095<br>FF104_19645
	Mannosylglycerate	**Mannosylglycerate hydrolase**	FF104_18580
		Mannosylglycerate transport	FF104_18575
	Trehalose transport and metabolism	**Trehalose PTS transporter**	FF104_19085
		**Alpha,alpha-phosphotrehalase**	FF104_19090
	N-acetylmuramic acid metabolism	**N-acetylmuramic acid 6-phosphate etherase**	FF104_19495
	Cellobiose transport and metabolism	Cellobiose PTS transporter (I)	FF104_19835<br>FF104_19840<br>FF104_19845
		Cellobiose PTS transporter (II)	FF104_19475
		Cellobiose PTS transporter subunit IIB	FF104_19490
		Cellobiose PTS transporter subunit IIC	FF104_18620<br>FF104_19485
		Cellobiase	FF104_19850<br>FF104_19480<br>FF104_18635
	Glycerol-3-phosphate transport	**Glycerol-3-phosphate ABC transporter**	FF104_20250<br>FF104_20255<br>FF104_20260
		Glycerol-3-phosphate ABC transporter	FF104_18715<br>FF104_18720<br>FF104_18725
		Glycerol-3-phosphate transporter, periplasmic-binding protein	FF104_19970

	Glucose transport	Glucose PTS transporter	FF104_20350
	Maltose metabolism	6-phospho-alpha-glucosidase	FF104_20355
	Arbutin metabolism	6-phospho-beta-glucosidase	FF104_20445
	Polysaccharide transport and metabolism		
	Xyloside metabolism	**Alpha-xylosidase**	FF104_18110
		**Xylose isomerase**	FF104_18205
		**Beta-xylosidase**	FF104_18640
		**Xylulokinase**	FF104_20830
	Glucoside transport and metabolism	**Beta-glucosidase**	FF104_18115
		PTS alpha-glucoside transporter subunit IIBC	FF104_18875
		PTS beta-glucoside transporter subunit EIIBCA	FF104_20450
		YcjR sugar phosphate isomerase/epimerase	FF104_19950
	Fucoside metabolism	**Alpha-L-fucosidase**	FF104_18125
	Alginate transport	**Alginate extracellular solute-binding protein**	FF104_18130
	Arabinogalactan metabolism	**Arabinogalactan endo-1,4-beta-galactosidase**	FF104_20240
		Beta-galactosidase	FF104_20245
	Central metabolism		
	Glycolysis	Fructose-6-phosphate aldolase	FF104_18035<br>FF104_18565<br>FF104_20820
		Phosphopyruvate hydratase	FF104_18260
	Pentose phosphate pathway	Transketolase	FF104_18040<br>FF104_20815
	Other		
	Lipid A metabolism (carbohydrate modification)	**NAD-dependent epimerase/dehydratase family protein involved in lipid A modification**	FF104_19565
		Polysaccharide deacetylase	FF104_18765<br>FF104_20110
	Other carbohydrate metabolism	**Sugar isomerase**	FF104_18205
		**ROK family transcriptional regulator**	FF104_19855
		**Beta-glucanase**	FF104_19860
		**Polysaccharide transport**	FF104_20265
		**MFS transporter, unknown substrate**	FF104_20335<br>FF104_20560<br>FF104_21235
		**Hypothetical protein**	FF104_21160
**H**	Coenzyme metabolism	**Ribokinase**	FF104_18010
		**UbiD family decarboxylase**	FF104_18450
		**UbiX family flavin prenyltransferase**	FF104_18455
		**2-dehydropantoate 2-reductase**	FF104_19010<br>FF104_19875
		**S-ribosylhomocysteine lyase**	FF104_20960
		tRNA-dihydrouridine synthase	FF104_18705<br>FF104_19995
		Cob(I)yrinic acid a,c-diamide synthase	FF104_19585
		Dihydrofolate reductase	FF104_20005
		Aminotransferase class III-fold pyridoxal phosphate-dependent enzyme	FF104_20805
	Coenzyme transport	**ABC-type Fe3+-siderophores transport system**	FF104_20305<br>FF104_21205
		PTS sugar transporter subunit IIB	FF104_21020
**I**	Lipid transport and metabolism	**Tannase B**	FF104_18625<br>FF104_20880
		**SDR family oxidoreductase**	FF104_21250
		Gluconate 5-dehydrogenase	FF104_19150
		Phosphatidic acid phosphatase type 2	FF104_21010
**J**	Translation, ribosomal structures and biogenesis	**GNAT family N-acetyltransferase**	FF104_18245<br>FF104_18255<br>FF104_19375<br>FF104_19520<br>FF104_20035
		**RNA 2',3'-cyclic phosphodiesterase**	FF104_19655
		Peptide deformylase	FF104_18140
		Ribosomal protein L11 methylase PrmA	FF104_19580
		GNAT family N-acetyltransferase	FF104_20105
		Threonine--tRNA ligase	FF104_21120
**K**	Transcription	**Helix-turn-helix transcriptional regulator**	FF104_18185<br>FF104_18375<br>FF104_19145<br>FF104_19175

		FF104_19380
		FF104_19725
		FF104_20010
		FF104_20200
		FF104_20330
		FF104_20605
		FF104_21110
		FF104_21185
		FF104_21230
	**MarR family transcriptional regulator**	FF104_18350
		FF104_19165
	**AraC family transcriptional regulator**	FF104_18585
		FF104_18630
		FF104_20045
	**MurR/RpiR family transcriptional regulator**	FF104_18570
		FF104_18610
		FF104_19045
		FF104_19450
		FF104_20340
		FF104_20345
		FF104_20925
		FF104_21135
	**LysR family transcriptional regulator**	FF104_18830
		FF104_19005
		FF104_19605
		FF104_21005
	**BlaI/MecI/CopY family transcriptional regulator**	FF104_18600
	**Peptidase M56**	FF104_18605
	**LuxR family transcriptional regulator**	FF104_18685
	**DeoR/GlpR transcriptional regulator**	FF104_18990
	**Protein kinase family protein**	FF104_19075
	**Trehalose operon repressor**	FF104_19095
	**GNAT family N-acetyltransferase**	FF104_19125
		FF104_19400
		FF104_20065
	**Zinc-binding hypothetical protein**	FF104_19205
	**MerR family transcriptional regulator**	FF104_19355
		FF104_21215
	**TetR/AcrR family transcriptional regulator**	FF104_19240
		FF104_19730
		FF104_19775
		FF104_20090
	**N-acetyltransferase**	FF104_19500
	**Aldo/keto reductase**	FF104_19675
		FF104_20915
	**Winged helix-turn-helix transcriptional regulator**	FF104_20375
	**Response regulator transcription factor**	FF104_21255
	MarR family transcriptional regulator	FF104_17935
		FF104_19220
	LacI family transcriptional regulator	FF104_18045
		FF104_18360
		FF104_18915
		FF104_19830
		FF104_19865
	LysR family transcriptional regulator	FF104_18220
		FF104_18445
		FF104_18700
		FF104_20140
	P-II family nitrogen regulator	FF104_18305
		FF104_18310
	DeoR/GlpR transcriptional regulator	FF104_18550
	MerR family transcriptional regulator	FF104_18690
		FF104_19510
	Cytochrome C551	FF104_18870
	FadR family transcriptional regulator	FF104_18945
	GNAT family N-acetyltransferase	FF104_19365
	Transcription elongation factor GreA	FF104_19640
	Winged helix-turn-helix transcriptional regulator	FF104_19765
	Glycerol-3-phosphate responsive antiterminator	FF104_19980
	Hypothetical protein	FF104_19985
	LytR/AlgR family DNA-binding response regulator	FF104_20215
	PRD domain-containing protein	FF104_20455

		Response regulator transcription factor	FF104_20405<br>FF104_20900
		Plasmid partition protein ParB	FF104_21090
L	Replication, recombination and repair	**ATP-dependent helicase**	FF104_18490
		**ATP-dependent endonuclease**	FF104_18485
		**DNA topoisomerase I**	FF104_18505
		**Protein-L-IsoD**	FF104_18885
		**RNA-directed DNA polymerase**	FF104_19210
		**NAD(+) diphosphatase, NudC**	FF104_19685
		**Tyrosine-type recombinase/integrase**	FF104_21085
		Exonuclease	FF104_19030<br>FF104_20870
		Tyrosine recombinase XerC	FF104_19315
		DEAD/DEAH box helicase	FF104_19600
		DNA polymerase III subunit alpha	FF104_20855
M	Cell wall biogenesis	**TQXZ domain-containing protein**	FF104_19670
		**Efflux RND transporter periplasmic adaptor subunit**	FF104_17920
		**Cell wall glycosyltransferase**	FF104_18170<br>FF104_18180
		**Murein hydrolase effector**	FF104_18935
		**Cyclopropane fatty acid methyltransferase**	FF104_18950
		**HlyD family efflux transporter periplasmic adaptor subunit**	FF104_20085
		**Exopolygalacturonate lyase**	FF104_20585
		**Silent information regulator protein**	FF104_20580
		**YqcG toxin**	FF104_20640
		**Hypothetical protein**	FF104_20685
		**YqcG toxin**	FF104_20700
		**Hypothetical protein**	FF104_20725
		N-acetyltransferase family protein	FF104_18815
		N-acetylmuramoyl-L-alanine amidase family protein	FF104_19190
		dTDP-4-dehydrorhamnose reductase	FF104_19505
N	Cell motility	**Plasmid partition protein ParM**	FF104_21100
		Flagellin	FF104_19260
		Hypothetical protein	FF104_19330
		Methyl-accepting chemotaxis protein	FF104_17985<br>FF104_18135<br>FF104_19335<br>FF104_20930
O	Post-translational modification, protein turnover, chaperones	**Flotillin family protein**	FF104_18665
		**Anhydro-N-acetylmuramic acid kinase**	FF104_19460
		**Peptidase U32**	FF104_20295
		**Glutathione peroxidase**	FF104_21030<br>FF104_21035
		Anaerobic ribonucleoside-triphosphate reductase activating protein	FF104_18050
		Peptidylpropyl isomerase	FF104_18335
		Peroxiredoxin	FF104_18435
P	Molybdate transport	**Molybdate ABC transporter (I)**	FF104_18265<br>FF104_18270<br>FF104_18275
		**Molybdate ABC transporter (II)**	FF104_18280<br>FF104_18295
		**Molybdate ABC transporter (III)**	FF104_19820<br>FF104_19825
	Cobalt transport	**Cobalt ABC transporter (I)**	FF104_21045<br>FF104_21050<br>FF104_21055<br>FF104_21060
		**Cobalt ABC transporter (II)**	FF104_20520<br>FF104_20525<br>FF104_20530
		**Cobalt ABC transporter, transmembrane component**	FF104_21150
	Taurine transport	**Taurine ABC transporter periplasmic component**	FF104_18975
	Toxic anion transport	**Toxic anion resistance protein**	FF104_19340
	Quaternary ammonium compound transport	**Quaternary ammonium compound efflux SMR transporter SugE**	FF104_20990
	Sodium transport	**Sodium/glutamate symporter**	FF104_21115
	Iron transport	Ferrous iron transport protein A	FF104_17990
		Ferrous iron transport protein B	FF104_17995
		FeoB-associated Cys-rich membrane protein	FF104_18000

		Iron ABC transporter	FF104_20310<br>FF104_20315<br>FF104_21190<br>FF104_21195<br>FF104_21200
	Formate transport	Formate transporter family protein	FF104_18650
	Magnesium transport	Magnesium transporter	FF104_18760
	Zinc transport	Zinc transporter ZupT	FF104_18800
	Potassium transport	Potassium transporter Kup	FF104_21170
	Sulfonate transport	ABC transporter ATP-binding protein<br>ABC transporter permease<br>ABC transporter permease subunit	FF104_18960<br>FF104_18965<br>FF104_18970
	Inorganic ion metabolism	CoA-disufide reductase	FF104_19265
	Inorganic phosphate transport	Na/Pi cotransporter family protein	FF104_19270
	Sugar transport (miscategorised)	**ABC transporter ATP-binding protein, galactoside transport**	FF104_18020
		**MFS transporter, galactoside transport**	FF104_18365
		ABC transporter ATP-binding protein, maltose transport	FF104_18710
		ABC transporter, maltose transport	FF104_19955<br>FF104_19960<br>FF104_19965
**Q**	Secondary metabolites biosynthesis and catabolism	**PucR family transcriptional regulator**	FF104_18210<br>FF104_19280<br>FF104_20810
		**Class I SAM-dependent methyltransferase**	FF104_18910
		**D-aminoacylase**	FF104_19920
		**Nicotinamidase**	FF104_19370<br>FF104_20195
**T**	Signal transduction	**Sensor histidine kinase**	FF104_17915<br>FF104_17975<br>FF104_18400<br>FF104_19425<br>FF104_20230<br>FF104_20275<br>FF104_20400<br>FF104_20885<br>FF104_20905<br>FF104_21065
		**PAS domain S-box protein**	FF104_18065<br>FF104_19060
		**Phosphodiesterase**	FF104_18745<br>FF104_18810
		**Crp/Fnr family transcriptional regulator**	FF104_19035
		**Serine/threonine protein phosphatase**	FF104_19635
		**GHKL domain-containing protein**	FF104_20210
		**Phosphatase**	FF104_20565<br>FF104_20715
		**Hypothetical protein**	FF104_20750
		**Hypothetical protein**	FF104_20765
		Carbon starvation protein A	FF104_18165
		Diguanylate cyclase	FF104_18795<br>FF104_19140
		Bifunctional diguanylate cyclase/phosphodiesterase	FF104_19015
		Methyl-accepting chemotaxis protein	FF104_19650
		Response regulator transcription factor	FF104_19430<br>FF104_20270
		OmpR family DNA-binding response regulator	FF104_17970<br>FF104_20235<br>FF104_20890
**U**	Secretion	**DHA2 family efflux MFS transporter permease subunit**	FF104_20075
**V**	Antibiotic efflux	**Efflux RND transporter**	FF104_17925
		**MATE family efflux transporter**	FF104_18865<br>FF104_18895<br>FF104_18920<br>FF104_19350<br>FF104_19710<br>FF104_20325
		**DHA2 family efflux MFS transporter**	FF104_20080
		**ABC transporter, bacitracin resistance**	FF104_20415
		ABC transporter, multidrug resistance	FF104_18145<br>FF104_18190<br>FF104_18195<br>FF104_18675

			FF104_18680<br>FF104_19225<br>FF104_19230<br>FF104_19940<br>FF104_19945<br>FF104_20220<br>FF104_20225<br>FF104_20390
	Antibiotic degradation	**Beta-lactamase**	FF104_17965<br>FF104_20595
		**AAC(3) family N-acetyltransferase**	FF104_18925
**X**	Transposons	**IS1182-like element ISClbu1 family transposase**	FF104_18100<br>FF104_18120
		**IS4 family transposase**	FF104_18410
		**IS3 family transposase**	FF104_18470<br>FF104_18475<br>FF104_19025
		**Transposase InsE**	FF104_18545
		**IS110 family transposase**	FF104_19810<br>FF104_20730
		**IS256 family transposase**	FF104_20180<br>FF104_20485
**Φ**	Nitrogen fixation	**NifH, Nitrogenase II**	FF104_18300
		**NifD, Nitrogenase molybdenum-iron protein subunit alpha**	FF104_18315
		**NifD, Nitrogenase molybdenum-iron protein subunit beta**	FF104_18320
		**NifE, Nitrogenase iron-molybdenum cofactor biosynthesis**	FF104_18325
		**NifD, NifN-B Nitrogenase**	FF104_18330
	Ethanolamine transport and metabolism	**Ethanolamine permease**	FF104_20435
		**Ethanolamine ammonia-lyase subunit EutC**	FF104_20425
		**Ethanolamine ammonia-lyase subunit EutB**	FF104_20430

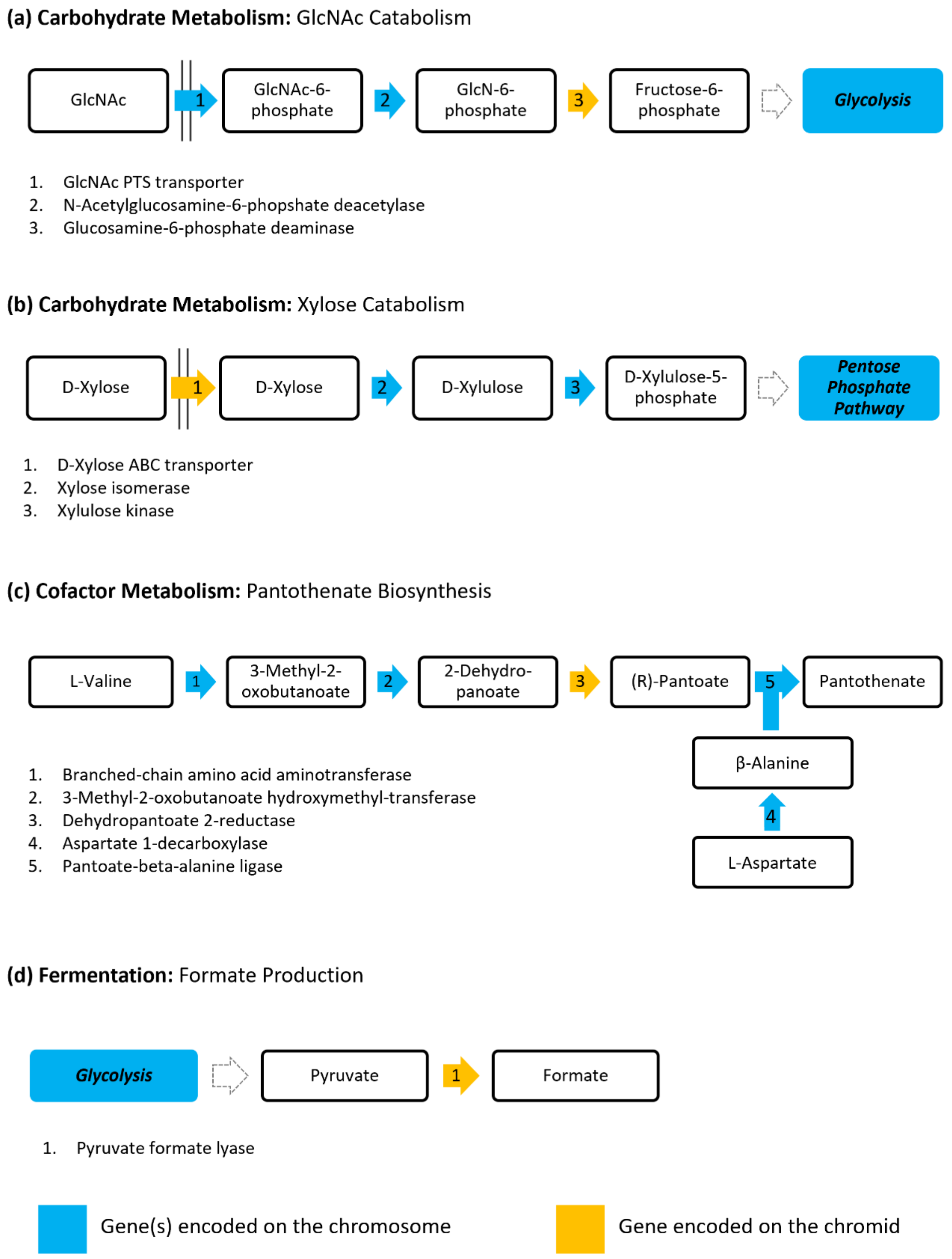

Fig. S2. Examples of ‘split’ metabolic pathways, in which the components of the pathway are encoded across both the chromid and the chromosome. (a) GlcNAc catabolic pathway. (b) Xylose catabolic pathway. (c) Pantothenate biosynthetic pathway. (d) Formate formation through the cleavage of pyruvate.

Table S5. COG categories, numbers of genes and relative gene frequencies for the core genes encoded on the chromosome and the chromid. COG categories have been ordered by the percentage change in the gene frequencies for each COG category on the chromid compared to the chromosome, from largest to smallest.

COG Category		Number of Genes		Number of Genes/Mb		Percentage enrichment of genes on the chromid (%)
**Code**	**Function**	**Chromosome**	**Chromid**	**Chromosome**	**Chromid**	
CE	Energy production and conversion/Amino acid transport and metabolism	0	2	0	2.5	∞
HP	Coenzyme transport and metabolism/Inorganic ion transport and metabolism	0	1	0	1.3	∞
EPG	Amino acid transport and metabolism/ Inorganic ion transport and metabolism/ Carbohydrate transport and metabolism	1	1	0.3	2.5	733
EM	Amino acid transport and metabolism/Cell wall/membrane/envelope biogenesis	1	1	0.3	1.3	333
GK	Carbohydrate transport and metabolism/Transcription	3	2	0.8	2.5	213
QT	Secondary metabolites biosynthesis, transport and catabolism/Signal transduction mechanisms	3	2	0.8	2.5	213
Q	Secondary metabolites biosynthesis, transport and catabolism	7	4	1.8	5	178
CH	Energy production and conversion/Coenzyme transport and metabolism	2	1	0.5	1.3	160
KQ	Transcription/Secondary metabolites biosynthesis, transport and catabolism	2	1	0.5	1.3	160
V	Defense mechanisms	46	22	11.8	27.5	133
KT	Transcription/Signal transduction mechanisms	12	5	3.1	6.3	103
K	Transcription	175	59	44.9	73.8	64
KLT	Transcription/Replication, recombination and repair/ Signal transduction mechanisms	3	1	0.8	1.3	63
T	Signal transduction mechanisms	82	24	21	30	43
IQ	Lipid transport and metabolism/Secondary metabolites biosynthesis, transport and catabolism	7	2	1.8	2.5	39
-	No assignment	255	67	65.4	83.8	28
C	Energy production and conversion	145	37	37.2	46.3	24
G	Carbohydrate transport and metabolism	186	46	47.7	57.5	21
P	Inorganic ion transport and metabolism	154	35	39.5	43.8	11
EG	Amino acid transport and metabolism/Carbohydrate transport and metabolism	11	2	2.8	2.5	-11
S	Function unknown	514	89	131.8	111.3	-16
O	Posttranslational modification, protein turnover, chaperones	61	7	15.6	8.8	-44
F	Nucleotide transport and metabolism	90	10	23.1	12.5	-46
M	Cell wall/membrane/envelope biogenesis	109	11	27.9	13.8	-51

NU	Cell motility/Intracellular trafficking, secretion and vesicular transport	11	1	2.8	1.3	-54
NT	Cell motility/Signal transduction mechanisms	33	3	8.5	3.8	-55
I	Lipid transport and metabolism	46	4	11.8	5	-58
N	Cell motility	23	2	5.9	2.5	-58
H	Coenzyme transport and metabolism	104	9	26.7	11.3	-58
E	Amino acid transport and metabolism	173	14	44.4	17.5	-61
L	Replication, recombination and repair	116	9	29.7	11.3	-62
D	Cell cycle control, cell division, chromosome partitioning	40	2	10.3	2.5	-76
J	Translation, ribosomal structure and biogenesis	170	8	43.6	10	-77
U	Intracellular trafficking, secretion and vesicular transport	26	1	6.7	1.3	-81
EH	Amino acid transport and metabolism/Coenzyme transport and metabolism	10	0	2.6	0	-∞
DZ	Cell cycle control, cell division, chromosome partitioning/Cytoskeleton	7	0	1.8	0	-∞
ET	Amino acid transport and metabolism/Signal transduction mechanisms	6	0	1.5	0	-∞
OU	Posttranslational modification, protein turnover, chaperones/Intracellular trafficking, secretion and vesicular transport	3	0	0.8	0	-∞
EJ	Amino acid transport and metabolism/Translation, ribosomal structure and biogenesis	2	0	0.5	0	-∞
EP	Amino acid transport and metabolism/Inorganic ion transport and metabolism	2	0	0.5	0	-∞
EQ	Amino acid transport and metabolism/Secondary metabolites biosynthesis, transport and catabolism	2	0	0.5	0	-∞
GM	Carbohydrate transport and metabolism/Cell wall/membrane/envelope biogenesis	2	0	0.5	0	-∞
HK	Coenzyme transport and metabolism/Transcription	2	0	0.5	0	-∞
BK	Chromatin structure and dynamics/Transcription	1	0	0.3	0	-∞
CO	Energy production and conversion/Posttranslational modification, protein turnover, chaperones	1	0	0.3	0	-∞
FG	Nucleotide transport and metabolism/Carbohydrate transport and metabolism	1	0	0.3	0	-∞
FJ	Nucleotide transport and metabolism/Translation, ribosomal structure and biogenesis	1	0	0.3	0	-∞
GIM	Carbohydrate transport and metabolism/Lipid transport and metabolism/Cell wall/membrane/envelope biogenesis	1	0	0.3	0	-∞
HJM	Coenzyme transport and metabolism/Translation, ribosomal structure and biogenesis/Cell	1	0	0.3	0	-∞

	wall/membrane/envelope biogenesis					
IM	Lipid transport and metabolism/Cell wall/membrane/envelope biogenesis	1	0	0.3	0	-∞
JM	Translation, ribosomal structure and biogenesis/Cell wall/membrane/envelope biogenesis	1	0	0.3	0	-∞
KOT	Transcription/Posttranslational modification, protein turnover, chaperones/Signal transduction mechanisms	1	0	0.3	0	-∞
LU	Replication, recombination and repair/Intracellular trafficking, secretion, and vesicular transport	1	0	0.3	0	-∞
MU	Cell wall/membrane/envelope biogenesis/Intracellular trafficking, secretion, and vesicular transport	1	0	0.3	0	-∞
NOU	Cell motility/Posttranslational modification, protein turnover, chaperones/Intracellular trafficking, secretion, and vesicular transport	1	0	0.3	0	-∞
PQ	Inorganic ion transport and metabolism/Secondary metabolites biosynthesis, transport and catabolism	1	0	0.3	0	-∞
UW	Intracellular trafficking, secretion and vesicular transport/Extracellular structures	1	0	0.3	0	-∞
HJ	Coenzyme transport and metabolism/Translation, ribosomal structure and biogenesis	1	0	0.3	0	-∞

Table S6. KEGG categories and gene frequencies for the core genes encoded on the chromosome and the chromid. KEGG categories have been ordered by the percentage change in the gene frequencies for each COG category on the chromid compared to the chromosome, from largest to smallest.

KEGG Supercategory	KEGG Category		Number of Genes/Mb		Percentage enrichment of genes on the chromid (%)
	Code	Functions	Chromosome	Chromid	
Metabolism	1.11	Xenobiotics biodegradation and metabolism	2.6	8.9	242
Environmental information processing	3.1	Membrane transport	8.8	15.2	73
Metabolism	1.4	Nucleotide metabolism	7	11.4	63
Metabolism	1.1	Carbohydrate metabolism	33.8	51.9	54
Cellular processes	4.4	Cellular community	4.9	6.3	29
Cellular processes	1.3	Lipid metabolism	9.4	8.9	-5
Environmental information processing	3.2	Signal transduction	6.2	5.1	-18
Cellular processes	4.5	Cell motility	5.2	3.8	-27
Metabolism	1.2	Energy metabolism	15.1	8.9	-41
Genetic information processing	2.4	Replication and repair	7.8	3.8	-51
Metabolism	1.6	Metabolism of other amino acids	5.2	2.5	-52
Metabolism	1.9	Metabolism of terpenoids and polyketides	3.1	1.3	-58
Metabolism	1.8	Metabolism of cofactors and vitamins	15.3	6.3	-59
Metabolism	1.1	Biosynthesis of other secondary metabolites	6.5	2.5	-62
Genetic information processing	2.3	Folding, sorting and degradation	3.4	1.3	-62
Cellular processes	4.2	Cell growth and death	3.6	1.3	-64
Genetic information processing	2.2	Translation	4.9	1.3	-73
Metabolism	1.5	Amino acid metabolism	29.6	7.6	-74
Metabolism	1.7	Glycan biosynthesis and metabolism	7.8	1.3	-83

Table S7. Genomes and metadata for each of the 178 *C. butyricum* strains used for the Roary gene/presence analysis, ordered by genome size from largest to smallest. Genomes were downloaded from NCBI on 22nd January 2025.

Strain	Isolation Source	Genome Assembly Level	Genome Size (Mb)	GenBank Accession	Reference
394	NEC case	Contig	5.29	GCA_963669105.1	(1)
359	NEC case	Contig	5.24	GCA_963668945.1	(1)
375	NEC case	Contig	5.23	GCA_963668975.1	(1)
354	NEC case	Contig	5.22	GCA_963668765.1	(1)
366	NEC case	Contig	5.22	GCA_963668745.1	(1)
352	NEC case	Contig	5.22	GCA_963668925.1	(1)
361	NEC case	Contig	5.2	GCA_963668805.1	(1)
393	NEC case	Contig	5.2	GCA_963669125.1	(1)
395	NEC case	Contig	5.2	GCA_963669095.1	(1)
379	NEC case	Contig	5.05	GCA_963669035.1	(1)
NOR 33234	NEC case, stool sample	Contig	4.92	GCA_000785185.1	(34)
YIM B08210	Paris polyphylla var. yunnanensis root	Contig	4.81	GCA_040813935.1	-
YIM B08196	Paris polyphylla var. yunnanensis root	Contig	4.80	GCA_040813795.1	-
J1100102st1_C2_J1100102_180507	Human stool sample	Scaffold	4.76	GCA_039837295.1	(35)
BoNT E BL5262	Botulism case, stool sample	Scaffold	4.76	GCA_000182605.1	-
CFSA3987	NEC case, stool sample	Complete	4.75	GCA_007992895.1	(36)
CFSA3989	NEC outbreak, surface swab	Complete	4.75	GCA_009650315.1	(36)
J1100102st1_B1_J1100102_180507	Human stool sample	Scaffold	4.74	GCA_039837335.1	(35)
DJ065	Industrial solvent production	Contig	4.74	GCA_013149575.1	(37)
J1100102st1_D3_J1100102_180507	Human stool sample	Scaffold	4.72	GCA_039837265.1	(35)
BIOML-A2	Human stool sample	Contig	4.72	GCA_009876455.1	(38)
BSD3178071175st2_D3_BSD3178071175_160912	Human intestine	Scaffold	4.71	GCA_028210725.1	(35)
DSM 10702	Pig intestine	Complete	4.71	GCA_014131795.1	(39)
CFSA-TJ-E	Botulism case, stool sample	Complete	4.7	GCA_024399875.1	(40)
C281E	Human stool sample	Scaffold	4.69	GCA_043845285.1	(41)
364	NEC case	Contig	4.68	GCA_963668825.1	(1)
349	NEC case	Contig	4.68	GCA_963668875.1	(1)
C287C	Human stool sample	Scaffold	4.68	GCA_043845015.1	(41)
300064	NEC case	Contig	4.68	GCA_002940805.1	-
376	NEC case	Contig	4.68	GCA_963668775.1	(1)
BL-5262-9RE	Botulism case	Contig	4.67	GCA_011019995.1	(1)
YIM B08153	Paris polyphylla var. yunnanensis root	Contig	4.67	GCA_040814915.1	-
C185C	Human stool sample	Scaffold	4.66	GCA_043845705.1	(41)
385	NEC case	Contig	4.66	GCA_963669085.1	(1)
H102020560	Botulism case	Contig	4.66	GCA_011010845.1	-
H102020561	Botulism case	Contig	4.66	GCA_011010795.1	-
YIM B08195	Paris polyphylla var. yunnanensis root	Contig	4.66	GCA_040814355.1	-
DJ046	Industrial solvent production	Contig	4.66	GCA_014137675.1	(37)
C185A	Human stool sample	Scaffold	4.66	GCA_043845825.1	(41)
365	NEC case	Contig	4.65	GCA_963668855.1	(1)
AF13-51	Human stool sample	Scaffold	4.65	GCA_027685445.1	(42)
363	NEC case	Contig	4.65	GCA_963668965.1	(1)
AF13-166.0	Human stool sample	Scaffold	4.65	GCA_027685535.1	(42)
60E.3	Human intestine	Scaffold	4.64	GCA_000371625.1	(42)
DJ064	Industrial solvent production	Contig	4.64	GCA_013149585.1	(37)
CDC_51208	Botulism case	Complete	4.64	GCA_001886875.1	(21)
D52st1_A4_D52t1_170925	Human intestine	Scaffold	4.64	GCA_028210695.1	(35)
4-1	Human stool sample	Complete	4.64	GCA_005145085.1	(43)
QXYZ514	Soil	Complete	4.64	GCA_026651935.1	(44)
YIM B08212	Paris polyphylla var. yunnanensis root	Contig	4.63	GCA_040813845.1	-
16-3	Human stool sample	Complete	4.63	GCA_013112415.1	(45)
DKU-11	Human stool sample	Complete	4.63	GCA_030389005.1	(46)
GBW-N1	Broiler tissue sample	Complete	4.63	GCA_041937605.1	-
DJ075	Industrial solvent production	Contig	4.63	GCA_014137745.1	(37)
KNU-L09	Human stool sample	Complete	4.63	GCA_001456065.2	(47)
YIM B08213	Paris polyphylla var. yunnanensis root	Contig	4.63	GCA_040814195.1	-
KZ-1886	Soil	Scaffold	4.63	GCA_024396935.1	-
YIM B08216	Paris polyphylla var. yunnanensis root	Contig	4.63	GCA_040814045.1	-

LV1	Shrimp intestine	Complete	4.63	GCA_027627495.1	(48)
YIM B08217	Paris polyphylla var. yunnanensis root	Contig	4.62	GCA_040814035.1	-
MCC 0233	Probiotics	Contig	4.62	GCA_030675955.1	-
YIM B08197	Paris polyphylla var. yunnanensis root	Contig	4.62	GCA_040814445.1	-
YIM B08173	Paris polyphylla var. yunnanensis root	Contig	4.62	GCA_040815235.1	-
YIM B08201	Paris polyphylla var. yunnanensis root	Contig	4.62	GCA_040814575.1	-
YIM B08203	Paris polyphylla var. yunnanensis root	Contig	4.62	GCA_040814585.1	-
YIM B08186	Paris polyphylla var. yunnanensis root	Contig	4.62	GCA_040814225.1	-
YIM B08185	Paris polyphylla var. yunnanensis root	Contig	4.62	GCA_040814595.1	-
YIM B08179	Paris polyphylla var. yunnanensis root	Contig	4.62	GCA_040814375.1	-
YIM B08158	Paris polyphylla var. yunnanensis root	Contig	4.62	GCA_040815325.1	-
YIM B08199	Paris polyphylla var. yunnanensis root	Contig	4.62	GCA_040813855.1	-
YIM B08183	Paris polyphylla var. yunnanensis root	Contig	4.62	GCA_040814805.1	-
JKY6D1	Pit mud	Complete	4.62	GCA_001465175.1	(49)
YIM B08156	Paris polyphylla var. yunnanensis root	Contig	4.62	GCA_040815355.1	-
YIM B08181	Paris polyphylla var. yunnanensis root	Contig	4.62	GCA_040814835.1	-
YIM B08163	Paris polyphylla var. yunnanensis root	Contig	4.62	GCA_040814795.1	-
YIM B08152	Paris polyphylla var. yunnanensis root	Contig	4.62	GCA_040815315.1	-
YIM B08172	Paris polyphylla var. yunnanensis root	Contig	4.62	GCA_040815215.1	-
YIM B08200	Paris polyphylla var. yunnanensis root	Contig	4.62	GCA_040814155.1	-
YIM B08208	Paris polyphylla var. yunnanensis root	Contig	4.62	GCA_040813885.1	-
YIM B08215	Paris polyphylla var. yunnanensis root	Contig	4.62	GCA_040814055.1	-
YIM B08155	Paris polyphylla var. yunnanensis root	Contig	4.62	GCA_040815415.1	-
CBM588	Human stool sample	Complete	4.61	GCA_030758275.1	(50)
AF13-10-6.0-r	Human stool sample	Scaffold	4.61	GCA_027685605.1	(42)
LY33	Pig intestine	Scaffold	4.61	GCA_020075635.1	-
HM-68	Chicken intestine	Contig	4.6	GCA_000878275.1	-
TOA	Probiotics	Complete	4.6	GCA_001646605.1	(51)
S-45-5	Stool sample	Complete	4.59	GCA_003315755.1	(52)
YIM B08175	Paris polyphylla var. yunnanensis root	Contig	4.59	GCA_040815005.1	-
C152A	Human stool sample	Scaffold	4.59	GCA_043846265.1	(41)
C154I	Human stool sample	Scaffold	4.58	GCA_043846025.1	(41)
4928STDY7387863	Human stool sample	Scaffold	4.58	GCA_902165875.1	-
DSM 107392	Mouse intestine	Contig	4.57	GCA_932751065.1	(53)
MGYG-HGUT-00014	Human intestine	Scaffold	4.57	GCA_902362255.1	-
1001713B170214_170313_A7	Human stool sample	Scaffold	4.57	GCA_015557845.1	(35)
1001713st1_G1_1001713B170214_170313	Human intestine	Scaffold	4.57	GCA_028210195.1	(35)
AM21-3LB	Human stool sample	Scaffold	4.56	GCA_027671605.1	(42)
372	NEC case	Contig	4.56	GCA_963668845.1	(1)
D33t1_170424_B3	Human stool sample	Scaffold	4.56	GCA_015670975.1	(35)
LCL-155	Foodborne botulism	Scaffold	4.55	GCA_024397005.1	-
AGR2140	Rumen	Scaffold	4.55	GCA_000424245.1	-
KZ-1890	Soil, pathogen	Scaffold	4.55	GCA_024396995.1	-
353	NEC case	Contig	4.55	GCA_963669045.1	(1)
380	NEC case	Contig	4.55	GCA_963668755.1	(1)
351	NEC case	Contig	4.55	GCA_963668735.1	(1)
GD1_1	Pit mud	Scaffold	4.55	GCA_019913025.1	(54)
YIM B08036	Paris polyphylla var. yunnanensis root	Contig	4.55	GCA_040815475.1	-
381	NEC case	Contig	4.54	GCA_963669145.1	(1)
1001311st1_D1_1001311H_170123	Human intestine	Scaffold	4.54	GCA_028210715.1	(35)
YIM B08164	Paris polyphylla var. yunnanensis root	Contig	4.54	GCA_040814845.1	-
1001287H_170206_H10	Human stool sample	Scaffold	4.54	GCA_015669495.1	(35)
5521	Botulism case	Contig	4.54	GCA_000171115.1	(55)
YIM B08147	Paris polyphylla var. yunnanensis root	Contig	4.54	GCA_040815395.1	-
YIM B08154	Paris polyphylla var. yunnanensis root	Contig	4.54	GCA_040815375.1	-
YIM B08184	Paris polyphylla var. yunnanensis root	Contig	4.54	GCA_040814635.1	-
1001311H_170123_H1	Human stool sample	Scaffold	4.54	GCA_015667735.1	(35)
AM32-13	Human stool sample	Scaffold	4.54	GCA_027670425.1	(42)
C230B	Human stool sample	Scaffold	4.54	GCA_043845495.1	(41)
AF25-25	Human stool sample	Scaffold	4.54	GCA_003459015.1	(42)
NBRC 3315	Unknown	Contig	4.53	GCA_007992855.1	-
DJ013	Industrial solvent production	Contig	4.53	GCA_014230505.1	(37)
DKU-01	Human stool sample	Contig	4.52	GCA_000355785.1	(56)
HYCB	Chicken intestine	Contig	4.52	GCA_013377675.1	-
378	NEC case	Contig	4.52	GCA_963668815.1	(1)
LCL-063	Botulism case, stool sample	Scaffold	4.51	GCA_024397055.1	-
386	NEC case	Contig	4.51	GCA_963669075.1	(1)
CC00973	Infant with IBD, mucosal biopsy	Scaffold	4.51	GCA_964242235.1	-

ATCC 43755	Botulism case	Contig	4.51	GCA_011017415.1	-
CLA-SR-H018	Human stool sample	Contig	4.5	GCA_040096815.1	(57)
382	NEC case	Contig	4.5	GCA_963669135.1	(1)
377	NEC case	Contig	4.5	GCA_963668895.1	(1)
UTH001	Blood sample, probiotic trial	Contig	4.5	GCA_042852705.1	(58)
YIM B08204	Paris polyphylla var. yunnanensis root	Contig	4.5	GCA_040814405.1	-
387	NEC case	Contig	4.49	GCA_963669055.1	(1)
CWBI1009	Anaerobic sludge	Contig	4.49	GCA_000949905.1	(59)
CBUT	Probiotics	Complete	4.49	GCA_018140655.1	(15)
C287A	Human stool sample	Scaffold	4.48	GCA_043845125.1	(41)
374	NEC case	Contig	4.48	GCA_963668795.1	(1)
Avi11	Red junglefowl intestine	Contig	4.48	GCA_030372205.1	(60)
355	NEC case	Contig	4.46	GCA_963669015.1	(1)
369	NEC case	Contig	4.46	GCA_963668985.1	(1)
371	NEC case	Contig	4.46	GCA_963668885.1	(1)
362	NEC case	Contig	4.46	GCA_963668835.1	(1)
368	NEC case	Contig	4.46	GCA_963668935.1	(1)
370	NEC case	Contig	4.46	GCA_963668955.1	(1)
357	NEC case	Contig	4.46	GCA_963668725.1	(1)
384	NEC case	Contig	4.46	GCA_963669115.1	(1)
367	NEC case	Contig	4.46	GCA_963669005.1	(1)
383	NEC case	Contig	4.46	GCA_963669065.1	(1)
350	NEC case	Contig	4.46	GCA_963668915.1	(1)
358	NEC case	Contig	4.45	GCA_963668905.1	(1)
NBRC 3858	Unknown	Contig	4.45	GCA_007992875.1	(61)
B111	Chicken intestine	Contig	4.44	GCA_947381465.1	(2)
356	NEC case	Contig	4.42	GCA_963669025.1	(1)
3W	Camel intestine	Contig	4.42	GCA_032465735.1	-
ET61	Red junglefowl intestine	Contig	4.41	GCA_030371725.1	(60)
373	NEC case	Contig	4.41	GCA_963668785.1	(1)
YIM B08145	Paris polyphylla var. yunnanensis root	Contig	4.4	GCA_040815445.1	-
YIM B08143	Paris polyphylla var. yunnanensis root	Contig	4.38	GCA_040815485.1	-
YIM B08176	Paris polyphylla var. yunnanensis root	Contig	4.38	GCA_040814985.1	-
YIM B08178	Paris polyphylla var. yunnanensis root	Contig	4.37	GCA_040814165.1	-
YIM B08166	Paris polyphylla var. yunnanensis root	Contig	4.37	GCA_040815045.1	-
YIM B08150	Paris polyphylla var. yunnanensis root	Contig	4.37	GCA_040815225.1	-
YIM B08220	Paris polyphylla var. yunnanensis root	Contig	4.37	GCA_040811415.1	-
YIM B08165	Paris polyphylla var. yunnanensis root	Contig	4.37	GCA_040815055.1	-
YIM B08149	Paris polyphylla var. yunnanensis root	Contig	4.37	GCA_040815285.1	-
YIM B08177	Paris polyphylla var. yunnanensis root	Contig	4.37	GCA_040815495.1	-
YIM B08207	Paris polyphylla var. yunnanensis root	Contig	4.37	GCA_040814395.1	-
YIM B08171	Paris polyphylla var. yunnanensis root	Contig	4.37	GCA_040815275.1	-
YIM B08144	Paris polyphylla var. yunnanensis root	Contig	4.37	GCA_040815435.1	-
YIM B08205	Paris polyphylla var. yunnanensis root	Contig	4.37	GCA_040814185.1	-
YIM B08182	Paris polyphylla var. yunnanensis root	Contig	4.37	GCA_040813805.1	-
YIM B08202	Paris polyphylla var. yunnanensis root	Contig	4.37	GCA_040814615.1	-
YIM B08209	Paris polyphylla var. yunnanensis root	Contig	4.37	GCA_040813835.1	-
YIM B08159	Paris polyphylla var. yunnanensis root	Contig	4.37	GCA_040815175.1	-
YIM B08221	Paris polyphylla var. yunnanensis root	Contig	4.36	GCA_040811425.1	-
YIM B08168	Paris polyphylla var. yunnanensis root	Contig	4.36	GCA_040815155.1	-
YIM B08174	Paris polyphylla var. yunnanensis root	Contig	4.35	GCA_040815185.1	-
JKT37	Palm oil mill effluent	Scaffold	4.22	GCA_003849835.1	(62)

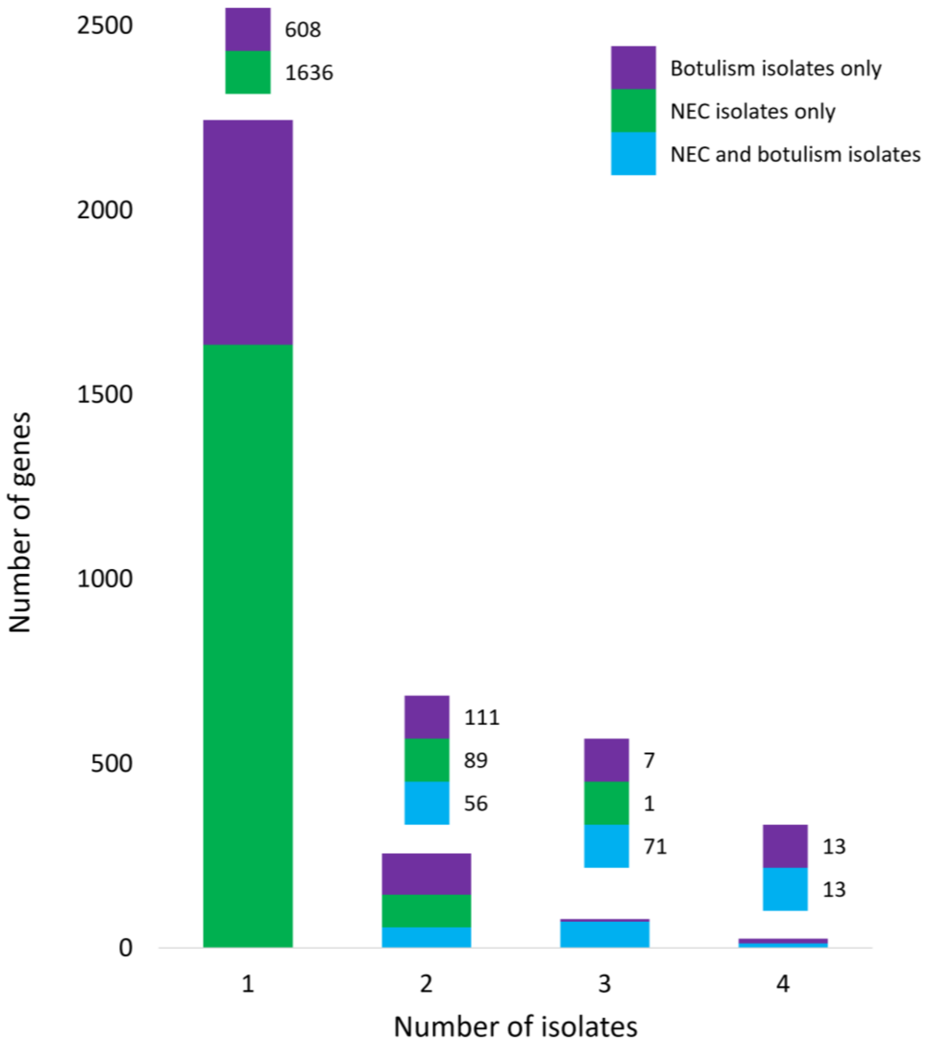

Fig S3. The distribution of genes that are present in the representative pathogenic isolates but absent from all non-pathogenic isolates. Boxes above each bar show the number of genes that are only found in either botulism or NEC isolates, in addition to those which can be found in isolates of both disease types.

Table S8. Genes that are conserved in the genomes of at least three of the twelve representative pathogenic *C. butyricum* strains, which include those present in each of the six conserved pathogen-associated gene clusters. Genes have been exemplified by those present in *C. butyricum* strains CDC_51208, BoNT BL5262, CFSA-TJ-E, LCL-155 and NOR 33234.

Pathogen-associated gene cluster	Predicted role	Genes	Predicted function	Present in botulism isolates	Present in NEC isolates
1	Botulinum toxin complex	NPD4_1437	OrfX3	✓	
		NPD4_1436	OrfX2		
		NPD4_1435	OrfX1		
		NPD4_1434	P-47 protein		
		NPD4_1433	NTNH, non-toxin nonhemagglutinin type A		
		NPD4_1432	Botulinum neurotoxin type E		
		NPD4_1431	HTH transcriptional regulator		
		NPD4_1430	RarA, replication-associated recombination protein A		
		NPD4_1429	Hypothetical protein		
		NPD4_1428	Hypothetical protein		
		NPD4_1427	Hypothetical protein		
		NPD4_1426	Hypothetical protein		
2	CPS biosynthesis	NPD4_767	dTDP-4-amino-4,6-dideoxy-D-glucose transaminase	✓	✓
		NPD4_766	CarB, carbamoyl phosphate large chain		
		NPD4_765	dTDP-glucose 4,6-dehydratase		
		NPD4_764	Metallophosphoesterase		
		NPD4_763	Membrane bound hypothetical protein		
		NPD4_762	ABC transporter, ATP-binding protein TagH		
		NPD4_761	Glycosyltransferase GT2		
		NPD4_760	ABC transporter, permease component TagG		
		NPD4_759	Glycosyltransferase GT1		
		NPD4_758	Glycosyltransferase GT2		
3	Flagellar glycosylation	NPD4_3101	FlgB, flagellar basal-body rod protein	✓	✓
		NPD4_3102	FlgL, flagellin		
		NPD4_3103	YjfB motility family protein		
		NPD4_3104	FlgL, flagellin		
		NPD4_3105	Maf, motility-associated factor		
		NPD4_3106	GlfM, Maf specificity factor		
		NPD4_3107	PseI, Pseudaminic acid synthase		
		NPD4_3108	Hypothetical protein		
		NPD4_3109	CDP-alcohol phosphatidyltransferase		
		NPD4_3110	2,3-bisphosphoglycerate-independent phosphoglycerate mutase		
		NPD4_3111	GlmU, *N*-acetylglucosamine-1-phosphate uridyltransferase		
		NPD4_3112	NDP-sugar epimerase		
		NPD4_3113	SAM-dependent methyltransferase		
		NPD4_3114	PseF, Pseudaminic acid cytidylyltransferase		
		NPD4_3115	PseG, UDP-2,4-diacetamido-2,4, 6-trideoxy-beta-L-altropyranose hydrolase		
		NPD4_3116	PseH, UDP-4-amino-4, 6-dideoxy-*N*-acetyl-beta-L-altrosamine *N*-acetyltransferase		
		NPD4_3117	Class I SAM-dependent methyltransferase		
		NPD4_3118	PseC, UDP-4-amino-4,6-dideoxy-*N*-acetyl-beta-L-altrosamine transaminase		
		NPD4_3119	PseB, UDP-*N*-acetylglucosamine 4,6-dehydratase		
		NPD4_3120	Maf, motility-associated factor		
		NPD4_3121	GT1 Glycosyltransferase		
		NPD4_3122	LegF, CMP-*N,N'*-diacetyllegionaminic acid synthase		
		NPD4_3123	LegG, GDP/UDP-*N,N'*-diacetylbacillosamine 2-epimerase		
		NPD4_3124	LegI, *N,N'*-diacetyllegionaminic acid synthase		
		NPD4_3125	LegH, GDP-4-amino-4,6-dideoxy-α-D-*N*-acetylglucosamine *N*-acetyltransferase		
		NPD4_3126	LegX, nucleotidyl transferase		
		NPD4_3127	LegC, PLP-dependent aminotransferase		
		NPD4_3128	Maf, motility-associated factor		

		NPD4_3129	LegB, NAD-dependent GDP-*N*-acetylglucosamine 4,6-dehydratase		
		NPD4_3130	FlgL, flagellin		
		NPD4_3131	DNA double-strand break repair Rad50 ATPase		
		NPD4_3132	FliD, flagellar hook associated protein D		
		NPD4_3133	FliS, flagellar protein		
		NPD4_3134	Hypothetical protein		
		NPD4_3135	FlaG, flagellar protein		
		NPD4_3136	CsrA global regulator protein		
		NPD4_3137	FliW, flagellar assembly factor		
**4**	Agr system, drug efflux	NPD4_977	ABC transporter, permease component	✓	✓
		NPD4_978	Macrolide ABC efflux transporter, ATPase component		
		NPD4_979	Phosphate regulon sensor protein PhoR		
		NPD4_980	Phosphate regulon transcriptional regulatory protein PhoB		
		NPD4_981	AgrA, accessory gene regulator protein A		
		NPD4_982	AgrC, accessory gene regulator protein C		
		NPD4_983	AgrD, accessory gene regulator protein D		
		NPD4_984	AgrB, accessory gene regulator protein B		
		NPD4_985	Hypothetical protein		
		NPD4_986	Multidrug ABC efflux transporter, ATPase component		
**5**	Copper resistance	NPD4_678	Hypothetical protein- secreted	✓	
		NPD4_679	CusR transcriptional regulator		
		NPD4_680	CusS sensor histidine kinase		
**6**	Drug efflux	NPD4_899	Secreted lipoprotein	✓	
		NPD4_900	Multidrug ABC efflux transporter, ATPase component		
		NPD4_901	Multidrug ABC efflux transporter, permease component		
		NPD4_902	Multidrug ABC efflux transporter, ATPase component		
		NPD4_903	Multidrug ABC efflux transporter, permease component		
	Other	CLP_0195	Lachnocin radical SAM maturase	✓	✓
		CLP_0196	S8 family serine peptidase	✓	✓
		CLP_0392	Xanthine dehydrogenase subunit D	✓	✓
		CLP_2582	Helix-turn-helix transcriptional regulator	✓	✓
		CLP_2583	Hypothetical protein	✓	✓
		CLP_2584	ParM	✓	✓
		CLP_2585	Hypothetical protein	✓	✓
		CLP_2586	Hypothetical protein	✓	✓
		CLP_2587	Hypothetical protein	✓	✓
		CLP_2629	Hypothetical protein	✓	✓
		CLP_3299	*N*-acetylmuramoyl-L-alanine amidase	✓	✓
		CLP_3303	Putative glycosyltransferase EpsD	✓	✓
		CLP_3485	Flagellar protein FlgN	✓	✓
		CLP_3486	Negative regulator of flagellin synthesis FlgM	✓	✓
		CLP_3497	Flagellar assembly protein FapA	✓	✓
		CLP_3761	Conserved hypothetical protein	✓	✓
		LJE39_07945	Tyrosine recombinase XerC	✓	✓
		LJE39_13565	Flagellin glycosyltransferase	✓	✓
		LJE39_13570	Flagellin lysine-*N*-methylase, FliB	✓	✓
		LJE39_13575	Flagellin	✓	✓
		LJE39_13580	Flagellar filament capping protein FliD	✓	✓
		LJE39_17465	Magnesium transporter MgtE	✓	✓
		LJE39_17490	Alpha/beta hydrolase	✓	✓
		LJE39_17500	Transposase	✓	✓
		LJE39_19970	IS1182 family transposase ISClbu1	✓	✓
		HNS01_10880	Hypothetical protein	✓	✓
		OA81_09770	Multidrug ABC transporter ATP-binding domain	✓	✓
		Unavailable	Hypothetical protein		✓

Table S9. Genes required for the uptake and catabolism of the six major monosaccharides present in mucin glycans, across ten *C. butyricum* strains. Genes highlighted in bold belong to incomplete genome assemblies, so their location on either the chromosome or the chromid could not be determined.

Sugar	Genes	*C. butyricum* strain									
		CSFA39897	CDC_51208	5521	BoNT E BL5262	BL-5262-9RE	DSM 10702	LV1	JKY6D1	TOA	4-1
L-fucose	*fucP*	-	NPD4_920	**CBY_1568**	**CLP_0124**	**FDE75_05295**	-	-	-	-	-
	fucU	-	NPD4_918	**CBY_1566**	**CLP_0122**	**FDE75_05305**	-	-	-	-	-
	fucI	-	NPD4_916	**CBY_1564**	**CLP_0120**	**FDE75_05315**	-	-	-	-	-
	fucK	-	NPD4_917	**CBY_1565**	**CLP_0121**	**FDE75_05310**	-	-	-	-	-
	fucA	-	NPD4_919	**CBY_1567**	**CLP_0123**	**FDE75_05300**	-	-	-	-	-
	fucO	-	NPD4_914	**CBY_1562**	**CLP_0118**	**FDE75_05325**	-	-	-	-	-
GlcNAc	*PTS IIBC*	EBL75_01175	NPD4_1033	**CBY_1684**	**CLP_0239**	**FDE75_13580**	FF104_03150	O4N14_14800	ATD26_13875	AZ909_13625	FBD76_04755
	PTS IIA	EBL75_20450	NPD4_567	**CBY_3798**	**CLP_4453**	**FDE75_14260**<br>**FDE75_20395**	FF104_00935	O4N14_16950	ATD26_16110	AZ909_15850	FBD76_06900
	nagA	EBL75_05795	NPD4_48	**CBY_3029**	**CLP_3866**	**FDE75_20400**	FF104_16260	O4N14_01675	ATD26_01660	AZ909_01575	FBD76_09500
	nagB	EBL75_01595*	NPD4_3707*	**CBY_0397**	**CLP_0863**	**FDE75_01245**	FF104_19050*	O4N14_21210*	ATD26_18225*	AZ909_18255*	FBD76_20215*
Galactose	*PTS IIC*	-	NPD4_91	**CBY_2987**	**CLP_3908**	**FDE75_01770**	FF104_16470	O4N14_01465	ATD26_01440	AZ909_01355	FBD76_09280
	PTS IIB	-	NPD4_92	**CBY_2986**	**CLP_3909**	**FDE75_01775**	FF104_16475	O4N14_01460	ATD26_01445	AZ909_01360	FBD76_09285
	PTS IIA	-	NPD4_93	**CBY_2985**	**CLP_3910**	**FDE75_01780**	FF104_16480	O4N14_01455	ATD26_01450	AZ909_01365	FBD76_09290
	lacA	EBL75_05535	NPD4_90	**CBY_2988**	**CLP_3907**	**FDE75_01765**	FF104_16465	O4N14_01470	ATD26_01455	AZ909_01370	FBD76_09295
	lacB	EBL75_05540	NPD4_89	**CBY_2989**	**CLP_3906**	**FDE75_01760**	FF104_16460	O4N14_01475	ATD26_01460	AZ909_01375	FBD76_09300
	lacC	EBL75_05550	NPD4_87	**CBY_2291**	**CLP_3904**	**FDE75_01750**	FF104_16450	O4N14_01485	ATD26_01470	AZ909_01385	FBD76_09310
	gatY	EBL75_05545	NPD4_88	**CBY_2290**	**CLP_3905**	**FDE75_01755**	FF104_16455	O4N14_01480	ATD26_01465	AZ909_01380	FBD76_09305
GalNAc	*PTS IIA*	EBL75_06190	NPD4_3436	**CBY_3478**	**CLP_3789**	**FDE75_20370**	FF104_15865	O4N14_02055	ATD26_02055	AZ909_01940	FBD76_09885
	PTS IIB	EBL75_06165	NPD4_3441	**CBY_3473**	**CLP_3794**	**FDE75_20395**	FF104_15890	O4N14_02030	ATD26_02030	AZ909_01915	FBD76_09860
	PTS IIC	EBL75_06170	NPD4_3440	**CBY_3474**	**CLP_3793**	**FDE75_20390**	FF104_15885	O4N14_02035	ATD26_02035	AZ909_01920	FBD76_09865
	PTS IID	EBL75_06175	NPD4_3439	**CBY_3475**	**CLP_3792**	**FDE75_20385**	FF104_15880	O4N14_02040	ATD26_02040	AZ909_01925	FBD76_09870
	agaA	EBL75_06160	NPD4_3442	**CBY_3472**	**CLP_3795**	**FDE75_20400**	FF104_15895	O4N14_02025	ATD26_02025	AZ909_01910	FBD76_09855
	agaS	EBL75_06185	NPD4_3437	**CBY_3477**	**CLP_3790**	**FDE75_20375**	FF104_15870	O4N14_02050	ATD26_02050	AZ909_01935	FBD76_09880
	lacC	EBL75_05550	NPD4_87	**CBY_2291**	**CLP_3904**	**FDE75_01750**	FF104_16450	O4N14_01485	ATD26_01470	AZ909_01385	FBD76_09310
	gatY	EBL75_06195	NPD4_3435	**CBY_3479**	**CLP_3788**	**FDE75_20365**	FF104_15860	O4N14_02060	ATD26_02060	AZ909_01945	FBD76_09890
Mannose	*PTS IIAB*	EBL75_00225*	NPD4_3898*	**CBY_1459**	**CLP_0645**	**FDE75_00180**	FF104_17945*	O4N14_20110*	ATD26_17245*	AZ909_17265*	FBD76_19160*
	PTS IIC	EBL75_00230*	NPD4_3897*	**CBY_1460**	**CLP_0646**	**FDE75_00185**	FF104_17950*	O4N14_20115*	ATD26_17250*	AZ909_17270*	FBD76_19165*
	PTS IID	EBL75_00235*	NPD4_3896*	**CBY_1461**	**CLP_0646**	**FDE75_00190**	FF104_17955*	O4N14_20120*	ATD26_17255*	AZ909_17275*	FBD76_19170*
	PTS IIA	EBL75_02370*	NPD4_4197*	**CBY_2123**	**CLP_3152**	**FDE75_19995**	FF104_19805*	O4N14_18765*	ATD26_18975*	AZ909_19010*	FBD76_17800*
	PTS IIB	EBL75_02365*	NPD4_4198*	**CBY_2126**	**CLP_3155**	**FDE75_20000**	FF104_19800*	O4N14_18760*	ATD26_18970*	AZ909_19005*	FBD76_17795*
	PTS IIC	EBL75_02360*	NPD4_4199*	**CBY_2124**	**CLP_3153**	**FDE75_20005**	FF104_19795*	O4N14_18755*	ATD26_18965*	AZ909_19000*	FBD76_17790*
	PTS IID	EBL75_02355*	NPD4_4200*	**CBY_2125**	**CLP_3154**	**FDE75_20010**	FF104_19790*	O4N14_18750*	ATD26_18960*	AZ909_18995*	FBD76_17785*
	manA	EBL75_18330	NPD4_1010	**CBY_1661**	**CLP_0216**	**FDE75_13695**	FF104_03020	O4N14_14910	ATD26_13990	AZ909_13740	FBD76_04865
Neu5Ac	*siaT*	EBL75_19155	NPD4_823	**CBY_0840**	**CLP_0031**	**FDE75_05755**	FF104_02255	O4N14_15710	ATD26_14790	AZ909_14545	FBD76_05665
	nanA	EBL75_19150	NPD4_824	**CBY_0841**	**CLP_0032**	**FDE75_05750**	FF104_02260	O4N14_15705	ATD26_14785	AZ909_14540	FBD76_05660
	nagK	EBL75_19165	NPD4_821	**CBY_0838**	**CLP_0029**	**FDE75_05765**	FF104_02245	O4N14_15720	ATD26_14800	AZ909_14555	FBD76_05675
	nanE	EBL75_19145	NPD4_825	**CBY_0838**<br>**CBY_1615**	**CLP_0033**	**FDE75_05745**	FF104_02265<br>FF104_02915	O4N14_15700	ATD26_14780	AZ909_14535	FBD76_05655
	nagA	EBL75_05795	NPD4_48	**CBY_3029**	**CLP_3866**	**FDE75_20400**	FF104_16260	O4N14_01675	ATD26_01660	AZ909_01575	FBD76_09500
	nagB	EBL75_01595*	NPD4_3707*	**CBY_0397**	**CLP_0863**	**FDE75_01245**	FF104_19050*	O4N14_21210*	ATD26_18225*	AZ909_18255*	FBD76_20215*

*Gene is located on the chromid.

Table S10. Genes which encode the components of the fucose catabolic pathway in *C. butyricum* 5521, and their most closely related orthologs in other species. The amino acid sequence identity between the protein encoded by each gene and its ortholog in *C. butyricum* is shown in brackets.

Gene	Species				
	Clostridium butyricum **5521**	***Clostridium perfringens*** **ATCC 13124**	***Clostridium baratii*** **771-14**	***Clostridium sardiniense*** **DSM 2632**	***Paraclostridium sordellii*** **JGS6956**
fucO	CBY_1562	CPF_1046 (88)	UC77_11170 (88)	JOC62_001813 (86)	JGS6956_05801 (82)
fucI	CBY_1564	CPF_1048 (92)	UC77_11160 (92)	JOC62_001815 (89)	JGS6956_05821 (89)
fucK	CBY_1565	CPF_1049 (75)	UC77_11155 (74)	JOC62_001816 (74)	JGS6956_05831 (76)
fucU	CBY_1566	CPF_1050 (75)	UC77_11150 (77)	JOC62_001817 (77)	JGS6956_05841 (77)
fucA	CBY_1567	CPF_1051 (85)	UC77_11145 (87)	JOC62_001818 (86)	JGS6956_05851 (87)
fucP	CBY_1568	CPF_1052 (77)	UC77_11140 (80)	JOC62_001819 (82)	JGS6956_05861 (79)

BLAST analyses show that the mean percentage amino acid identity between nine *C. butyricum* proteins and their orthologs in four closely related *Clostridium* species ranges from 71-90%. The highest average amino acid identity of 90 ± 3.1% (mean ± SD), and amino acid sequence similarity of 95 ± 2.2%, can be observed between the nine pairs of orthologs from *C. butyricum* and *C. saccharobutylicum*, which suggests that *C. saccharobutylicum* is the closest relative to *C. butyricum*.

Table S11. The amino acid sequence identity (bold) and similarity between nine genes from *C. butyricum* DSM 10702, and their orthologs in the type strains of four closely related *Clostridium* species.

COG Code	Gene	*C. butyricum* DSM 10702	Amino acid identity/similarity (%)							
			C. felsineum DSM 794		*C. beijerinckii* NCIMB 8052		*C. saccharoper-butylacetonicm* N1-4(HMT)		*C. saccharo-butylicum* DSM 13864	
D	GTPase ObgE/CgtA	FF104_15145	**70**	86	**87**	94	**87**	94	**91**	95
F	Guanylate kinase	FF104_05425	**70**	85	**86**	93	**84**	94	**90**	96
G	Phosphoglycerate kinase	FF104_14905	**80**	88	**95**	97	**95**	96	**94**	96
J	Ribosome-recycling factor	FF104_05680	**66**	84	**88**	96	**89**	96	**90**	96
K	Transcription termination/antitermination protein NusA	FF104_05725	**73**	89	**90**	95	**88**	94	**90**	96
L	Chromosomal replication initiator protein DnaA	FF104_00005	**76**	86	**93**	97	**92**	96	**93**	96
O	Trigger factor	FF104_12405	**56**	77	**88**	95	**82**	93	**86**	95
R	GTPase Der	FF104_05385	**71**	86	**87**	96	**92**	96	**93**	97
U	Protein translocase subunit SecA	FF104_15420	**74**	87	**89**	95	**82**	89	**84**	89

.

It was noted that the singular chromosome present in *C. saccharobutylicum* is a similar size to the combined size of the chromosome and chromid in *C. butyricum* (5.08 ± 0.07 Mb and 4.64 ± 0.06 Mb, respectively). As *C. saccharobutylicum* is the closest relative to *C. butyricum*, it was considered whether the chromid may have formed from the schism of a *C. saccharobutylicum* chromosome. However, the Mauve alignment shows that the chromid of *C. butyricum* is not homologous to a region of the *C. saccharobutylicum* chromosome, which may have supported the 'schism hypothesis' (20). Instead, regions of homology are distributed across the chromosome.

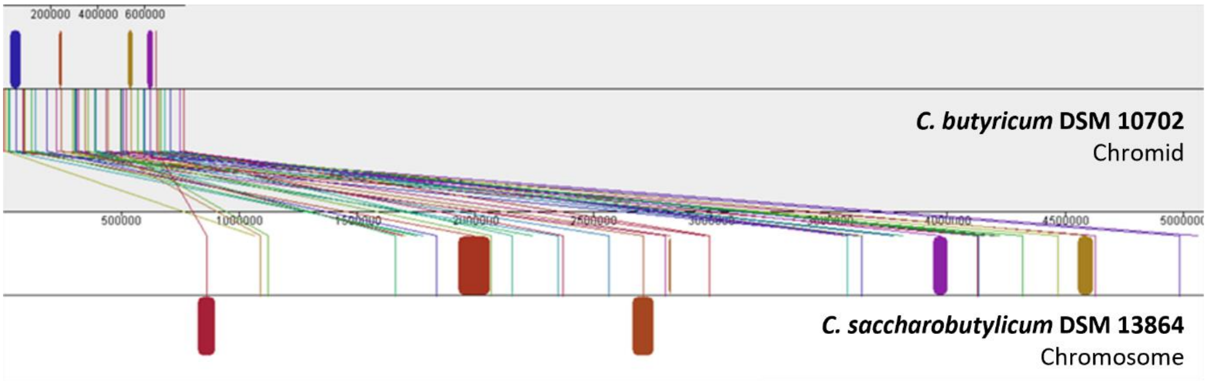

Fig. S4. Mauve alignment of the chromosome of *C. saccharobutylicum* DSM 13864 and the chromid of *C. butyricum* DSM 10702.

REFERENCES

1. Sartor C, Mikrat Y, Grandvuillemin I, Caputo A, Ligi I, Chanteloup A, *et al.* Investigating transmission patterns among preterm neonates during an outbreak of necrotizing enterocolitis related to *Clostridium butyricum* using whole-genome sequencing. *J Hosp Infect.* 2024;152:21–7.

2. Rios-Galicia B, Sáenz JS, Yergaliyev T, Roth C, Camarinha-Silva A, Seifert J. Novel taxonomic and functional diversity of eight bacteria from the upper digestive tract of chicken. *Int J Syst Evol Microbiol.* [Internet]. 202;74(1). Available from: https://www.microbiologyresearch.org/docserver/fulltext/ijsem/74/1/ijsem006210.pdf?expires=1743599249&id=id&accname=sgid025303&checksum=5BA05A681F1978D52ADD454ED53FD4A9

3. van der Lelie D, Oka A, Taghavi S, Umeno J, Fan T-J, Merrell KE, *et al.* Rationally designed bacterial consortia to treat chronic immune-mediated colitis and restore intestinal homeostasis. *Nat Commun.* 2021;12(1):3105.

4. Bruce T, Leite FG, Tschoeke DA, Miranda M, Pereira N Jr, Valle R, *et al.* Exploring the genome of a butyric acid producer, *Clostridium butyricum* INCQS635. *Genome Announc.* [Internet]. 2014;2(6). [cited 2025 Apr 2]. Available from: https://pubmed.ncbi.nlm.nih.gov/25414496/

5. Tanwar AS, Shruptha P, Jnana A, Brand A, Ballal M, Satyamoorthy K, *et al.* Emerging pathogens in planetary health and lessons from comparative genome analyses of three Clostridia species. *OMICS.* 2023;27(6):247–59.

6. Cassir N, Benamar S, Khalil JB, Croce O, Saint-Faust M, Jacquot A, *et al. Clostridium butyricum* Strains and Dysbiosis Linked to Necrotizing Enterocolitis in Preterm Neonates. *Clin Infect Dis*. 2015;61(7):1107–15. Available from: https://academic.oup.com/cid/article/61/7/1107/289593

7. Zhou J-J, Shen J-T, Jiang L-L, Sun Y-Q, Mu Y, Xiu Z-L. Selection and characterization of an anaerobic microbial consortium with high adaptation to crude glycerol for 1,3-propanediol production. *Appl Microbiol Biotechnol*. 2017;101(15):5985–96.

8. Sulthana A, Thorramamidi A, Lakshmi SG, Madempudi RS. Whole-genome shotgun sequencing and characterization of probiotic strain *Clostridium butyricum* UBCB 70 to assess its safety. *Microbiol Resour Announc*. 2019;8(5):e01732-18.

9. Yang Y, Shao Y, Pei C, Liu Y, Zhang M, Zhu X, *et al.* Pangenome analyses of *Clostridium butyricum* provide insights into its genetic characteristics and industrial application. *Genomics*. [Internet]. 2024;116(3). Available from: https://pubmed.ncbi.nlm.nih.gov/38703968/

10. Pei Z, Liu Y, Yi Z, Liao J, Wang H, Zhang H, *et al.* Diversity within the species *Clostridium butyricum*: pan-genome, phylogeny, prophage, carbohydrate utilization, and antibiotic resistance. *J Appl Microbiol.* [Internet]. 2023;134(7). Available from: https://pubmed.ncbi.nlm.nih.gov/37349950/

11. Zou W, Ye G, Zhang K, Yang H, Yang J. Analysis of the core genome and pangenome of *Clostridium butyricum. Genome*. 2021;64(1):51–61.

12. Poehlein A, Hartwich K, Krabben P, Ehrenreich A, Liebl W, Dürre P, *et al.* Complete genome sequence of the solvent producer *Clostridium saccharobutylicum* NCP262 (DSM 13864). *Genome Announc*. 2013;1(6):e00997-13.

13. Poehlein A, Solano JDM, Flitsch SK, Krabben P, Winzer K, Reid SJ, *et al.* Microbial solvent formation revisited by comparative genome analysis. *Biotechnol Biofuels*. 2017;10(1):58.

14. Sedlar K, Kolek J, Provaznik I, Patakova P. Reclassification of non-type strain *Clostridium pasteurianum* NRRL B-598 as *Clostridium beijerinckii* NRRL B-598. *J Biotechnol*. 2017;244:1–3.

15. Perraudeau F, McMurdie P, Bullard J, Cheng A, Cutcliffe C, Deo A, *et al.* Improvements to postprandial glucose control in subjects with type 2 diabetes: a multicenter, double blind, randomized placebo-controlled trial of a novel probiotic formulation. *BMJ Open Diabetes Res Care.* 2020;8(1):e001319.

16. Wang Y, Li X, Mao Y, Blaschek HP. Single-nucleotide resolution analysis of the transcriptome structure of *Clostridium beijerinckii* NCIMB 8052 using RNA-Seq. *BMC Genomics*. 2011;12(1):479.

17. Noar J, Makwana ST, Bruno-Bárcena JM. Complete genome sequence of solvent-tolerant *Clostridium beijerinckii* strain SA-1. *Genome Announc.* [Internet]. 2014;2(6). [cited 2025 Apr 2]. Available from: https://journals.asm.org/doi/10.1128/genomea.01310-14

18. Sedlar K, Nykrynova M, Bezdicek M, Branska B, Lengerova M, Patakova P, *et al.* Diversity and evolution of *Clostridium beijerinckii* and complete genome of the type strain DSM 791T. *Processes (Basel)*. 2021;9(7):1196.

19. Poehlein A, Krabben P, Dürre P, Daniel R. Complete genome sequence of the solvent producer *Clostridium saccharoperbutylacetonicum* strain DSM 14923. *Genome Announc.* [Internet]. 2014;2(5). Available from: https://journals.asm.org/doi/pdf/10.1128/genomea.01056-14?download=true

20. Harrison PW, Lower RPJ, Kim NKD, Young JPW. Introducing the bacterial "chromid": Not a chromosome, not a plasmid. *Trends Microbiol.* 2010;18(4):141–8.

21. Halpin JL, Hill K, Johnson SL, Bruce DC, Brian Shirey T, Dykes JK, *et al.* Finished Whole-Genome Sequences of *Clostridium butyricum* Toxin Subtype E4 and *Clostridium baratii* Toxin Subtype F7 Strains. *Genome Announc.* [Internet]. 2017;5(29). Available from: https://pubmed.ncbi.nlm.nih.gov/28729254/

22. Hahnke S, Wibberg D, Tomazetto G, Pühler A, Klocke M, Schlüterb A. Whole genome sequence of *Clostridium bornimense* strain M2/40 isolated from a lab-scale mesophilic two-phase biogas reactor digesting maize silage and wheat straw. *J Biotechnol*. 2014;184:199–200.

23. Bao G, Wang R, Zhu Y, Dong H, Mao S, Zhang Y, *et al.* Complete genome sequence of *Clostridium acetobutylicum* DSM 1731, a solvent-producing strain with multireplicon genome architecture. *J Bacteriol.* 2011;193(18):5007–8.

24. Cambridge JM, Blinkova AL, Salvador Rocha EI, Bode Hernández A, Moreno M, Ginés-Candelaria E, *et al.* Genomics of *Clostridium taeniosporum*, an organism which forms endospores with ribbon-like appendages. *PLoS One.* 2018;13(1):e0189673.

25. Jeong C-G, Seo B-J, Nazki S, Jung BK, Khatun A, Yang M-S, *et al.* Characterization of *Clostridium novyi* isolated from a sow in a sudden death case in Korea. *BMC Vet Res*. 2020;16(1):127.

26. Bruggemann H, Baumer S, Fricke WF, Wiezer A, Liesegang H, Decker I, *et al.* The genome sequence of *Clostridium tetani*, the causative agent of tetanus disease. *Proc Natl Acad Sci USA*. 2003;100(3):1316–21.

27. Lee J, Jang Y-S, Han M-J, Kim JY, Lee SY. Deciphering *Clostridium tyrobutyricum* metabolism based on the whole-genome sequence and proteome analyses. *MBio*. 2016;7(3):e00743-16.

28. Li N, Yang J, Chai C, Yang S, Jiang W, Gu Y. Complete genome sequence of *Clostridium carboxidivorans* P7(T), a syngas-fermenting bacterium capable of producing long-chain alcohols. *J Biotechnol.* 2015;211:44–5.

29. Sebaihia M, Peck MW, Minton NP, Thomson NR, Holden MTG, Mitchell WJ, *et al.* Genome sequence of a proteolytic (Group I) *Clostridium botulinum* strain Hall A and comparative analysis of the clostridial genomes. *Genome Res*. 2007;17(7):1082–1092. Available from: http://dx.doi.org/10.1101/gr.6282807

30. Jaakkola K, Virtanen K, Lahti P, Keto-Timonen R, Lindström M, Korkeala H. Comparative genome analysis and spore heat resistance assay reveal a new component to population structure and genome epidemiology within *Clostridium perfringens* enterotoxin-carrying isolates. *Front Microbiol*. 2021;12:717176.

31. Poehlein A, Bengelsdorf FR, Schiel-Bengelsdorf B, Gottschalk G, Daniel R, Dürre P. Complete genome sequence of Rnf- and cytochrome-containing autotrophic acetogen *Clostridium aceticum* DSM 1496. *Genome Announc*. 2015;3(4):e00786-15.

32. McGlinchey AS, Zepeda-Rivera MA, Stepanovica M, Baryiames AA, Jones DS, LaCourse KD, *et al.* Complete genome sequence of *Clostridium cadaveris* IFB3C5, isolated from a human colonic adenocarcinoma. *Microbiol Resour Announc*. 2022;11(3):e0113521.

33. Thomas P, Semmler T, Eichhorn I, Lübke-Becker A, Werckenthin C, Abdel-Glil MY, *et al.* First report of two complete *Clostridium* chauvoei genome sequences and detailed in silico genome analysis. *Infect Genet Evol.* 2017;54:287–98.

34. Kwok JSL, Ip M, Chan T-F, Lam W-Y, Tsui SKW. Draft genome sequence of *Clostridium butyricum* strain NOR 33234, isolated from an elderly patient with diarrhea. *Genome Announc.* [Internet]. 2014;2(6). [cited 2025 Apr 2]. Available from: https://pubmed.ncbi.nlm.nih.gov/25540356/

35. Chen-Liaw A, Aggarwala V, Mogno I, Haifer C, Li Z, Eggers J, *et al.* Gut microbiota strain richness is species specific and affects engraftment. *Nature*. 2025;637(8045):422–9.

36. Dong Y, Li Y, Zhang DBA, Nguyen S, Maheshwari N, Hu Y, *et al.* Epidemiological and genetic characterization of *Clostridium butyricum* cultured from neonatal cases of necrotizing enterocolitis in China. *Infect Control Hosp Epidemiol*. 2020;41(8):900–907. Available from: https://doi.org/10.1017/ice.2019.289

37. Liew FE, Nogle R, Abdalla T, Rasor BJ, Canter C, Jensen RO, *et al.* Carbon-negative production of acetone and isopropanol by gas fermentation at industrial pilot scale. *Nat Biotechnol*. 2022;40(3):335–44.

38. Poyet M, Groussin M, Gibbons SM, Avila-Pacheco J, Jiang X, Kearney SM, *et al.* A library of human gut bacterial isolates paired with longitudinal multiomics data enables mechanistic microbiome research. *Nat Med*. 2019;25(9):1442–52.

39. Xin B, Tao F, Wang Y, Gao C, Ma C, Xu P. Genome Sequence of *Clostridium butyricum* Strain DSM 10702, a Promising Producer of Biofuels and Biochemicals. *Genome Announc*. 2013;1(4):563–76.

40. Dong Y, Wang W, Jiang T, Xu J, Li F. Whole genome sequencing of *Clostridium butyricum* that caused the first infant botulism in China. *Disease Surveillance*. 2022;(1):38–44.

41. Dalby MJ, Kiu R, Serghiou IR, Miyazaki A, Acford-Palmer H, Tung R, *et al.* Faecal microbiota and cytokine profiles of rural Cambodian infants linked to diet and diarrhoeal episodes. *NPJ Biofilms Microbiomes*. 2024;10(1):85.

42. Lin X, Hu T, Chen J, Liang H, Zhou J, Wu Z, *et al.* The genomic landscape of reference genomes of cultivated human gut bacteria. *Nat Commun*. 2023;14(1):1663.

43. Bang M-S, Jeong H-W, Lee Y-J, Lee S-C, Lee GS, Kim S, *et al.* Complete Genome Sequence of *Clostridium butyricum* Strain DKU_*butyricum* 4-1, Isolated from Infant Feces. *Microbiol Resour Announc*. [Internet]. 2020;9(10). Available from: https://www.ncbi.nlm.nih.gov/pmc/articles/PMC7171215/

44. Yang M, Zabed HM, Yun J, Zhang G, Qi ·. Xianghui. The Draft Genome Sequence of *Clostridium butyricum* QXYZ514, a Potent Bacterium for Converting Glycerol into Fuels and Bioproducts in the Waste-Based Biorefinery. *Curr Microbiol*. 2020;77:3371–6.

45. Shin J-I, Bang M-S, Lee G-S, Kim H-N, Oh C-H. Draft Genome Sequence of *Clostridium butyricum* Strain 16-3, Isolated from Neonatal Feces. *American Society for Microbiology*. 2022;11(8).

46. Mo S. Complete genome sequence of *Clostridium butyricum* DKU-11, isolated from healthy infant feces. *Microbiol Resour Announc*. 2024;13(6):e0003724.

47. Shin J, Song Y, Jeong Y, Cho BK. Analysis of the core genome and pan-genome of autotrophic acetogenic bacteria [Internet]. *Frontiers in Microbiology*. 2016;7. Available from: http://dx.doi.org/10.3389/fmicb.2016.01531

48. Wang Q, Li W, Liu H, Tan B, Dong X, Chi S, *et al.* The isolation, identification, whole-genome sequencing of *Clostridium butyricum* LV1 and its effects on growth performance, immune response, and disease-resistance of *Litopenaeus vannamei*. *Microbiol Res*. 2023;272:127384.

49. Li C, Wang Y, Xie G, Peng B, Zhang B, Chen W, *et al.* Complete genome sequence of *Clostridium butyricum* JKY6D1 isolated from the pit mud of a Chinese flavor liquor-making factory. *J Biotechnol*. 2016;220:23–4.

50. Wood L, Omorotionmwan BB, Blanchard AM, Dowle A, Bishop AL, Griffin R. Characterisation of the butyrate production pathway in probiotic MIYAIRI588 by a combined whole genome-proteome approach. *bioRxiv*. 2023;2023:554021.

51. Isono A, Katsuno T, Sato T, Nakagawa T, Kato Y, Sato N, *et al.* *Clostridium butyricum* TO-A culture supernatant downregulates TLR4 in human colonic epithelial cells. *Digestive Diseases and Sciences*. 2007;52(11):2963–71.

52. Chathuranga K, Shin Y, Uddin MB, Paek J, Chathuranga WAG, Seong Y, *et al.* The novel immunobiotic *Clostridium butyricum* S-45-5 displays broad-spectrum antiviral activity in vitro and in vivo by inducing immune modulation. *Front Immunol*. 2023;14:1242183.

53. Afrizal A, Jennings SAV, Hitch TCA, Riedel T, Basic M, Panyot A, *et al.* Enhanced cultured diversity of the mouse gut microbiota enables custom-made synthetic communities. *Cell Host Microbe*. 2022;30(11):1630-1645.e25.

54. Luo H, Li T, Zheng J, Zhang K, Qiao Z, Luo H, *et al.* Isolation, identification, and fermentation medium optimization of a caproic acid-producing *Enterococcus casseliflavus* strain from pit mud of Chinese strong flavor baijiu ecosystem. *Pol J Microbiol*. 2022;71(4):563–75.

55. Hassan KA, Elbourne LDH, Tetu SG, Johnson EA, Paulsen IT. Genome sequence of the neurotoxigenic *Clostridium butyricum* strain 5521. *Genome Announc*. [Internet]. 2014;2(3). Available from: http://dx.doi.org/10.1128/genomeA.00632-14

56. Mo S, Kim BS, Yun SJ, Lee JJ, Yoon SH, Oh CH. Genome sequencing of *Clostridium butyricum* DKU-01, isolated from infant feces. *Gut Pathog*. [Internet]. 2015;7(1). Available from: https://www.ncbi.nlm.nih.gov/pmc/articles/PMC4390090/

57. Hitch TCA, Masson JM, Pauvert C, Bosch J, Nüchtern S, Treichel N, *et al.* Broad diversity of human gut bacteria accessible via a traceable strain deposition system [Internet]. *bioRxiv*. 2024;599854. [cited 2025 Apr 3]. Available from: https://www.biorxiv.org/content/10.1101/2024.06.20.599854v2.abstract

58. Sada RM, Motooka D, Kutsuna S, Hamaguchi S, Yamamoto G, Ueda A. 233. All *Clostridium butyricum* strains isolated from blood cultures were derived from probiotics: a single-centre retrospective study. *Open Forum Infect Dis*. 2023;10(Supplement_2):ofad500.306.

59. Calusinska M, Hamilton C, Monsieurs P, Mathy G, Leys N, Franck F, *et al.* Genome-wide transcriptional analysis suggests hydrogenase- and nitrogenase-mediated hydrogen production in *Clostridium butyricum* CWBI 1009. *Biotechnol Biofuels*. 2015;8(1):1–16.

60. Medvecky M, Cejkova D, Polansky O, Karasova D, Kubasova T, Cizek A, *et al.* Whole genome sequencing and function prediction of 133 gut anaerobes isolated from chicken caecum in pure cultures. *BMC Genomics*. 2018;19(1):561.

61. Sada RM, Matsuo H, Motooka D, Kutsuna S, Hamaguchi S, Yamamoto G, *et al.* *Clostridium butyricum* bacteremia associated with probiotic use, japan. *Emerg Infect Dis*. 2024;30(4):665–71.

62. Tee ZK, Jahim JM, Tan JP, Kim BH. Preeminent productivity of 1,3-propanediol by *Clostridium butyricum* JKT37 and the role of using calcium carbonate as pH neutraliser in glycerol fermentation. *Bioresour Technol*. 2017;233:296–304.
